# Emotional Congruence with Children: An Empirical Examination of Different Models in Men with a History of Sexually Offending Against Children

**DOI:** 10.1177/10790632231172160

**Published:** 2023-06-05

**Authors:** Julia M. Fraser, Kelly M. Babchishin, L. Maaike Helmus

**Affiliations:** 1 Department of Psychology, 6339 Carleton University, Ottawa, ON, Canada; 2 Department of Criminology, 1763 Simon Fraser University, Burnaby, British Columbia

**Keywords:** emotional congruence with children, sexual offending against children, sexual recidivism, sexual deviance, pedophilia

## Abstract

Emotional congruence with children (ECWC) is a psychologically meaningful risk factor for sexual offending against children (SOC). Based on previous research and theory, three models have been proposed to explain ECWC: Blockage, Sexual Domain, and Psychological Immaturity. Using structural equation modelling in a routine correctional sample of men adjudicated for sexual offences (*n* = 983), we found little support for all three of these models. Instead, we found that atypical sexual interests, alone, best explained ECWC, with a moderate relationship to ECWC. Using the predictors associated with each of the three models of ECWC, we identified three classes of men with a history of SOC who are high in ECWC using latent class analyses (*n* = 377). These three classes generally did not replicate the three models of ECWC. We instead propose three subgroups of men with histories of SOC who are high in ECWC, characterized respectively by: relationship deficits; youth and loneliness; and high sexual and general criminality. High levels of ECWC are predictive of a higher risk of sexual recidivism, regardless of class association; however, these subgroups are differentially at risk for some types of recidivism. Our findings suggest that ECWC is a multi-faceted construct, which is still not well understood.

## Introduction

Emotional congruence with children (ECWC) is a psychologically meaningful risk factor for sexual offending against children (Mann et al., 2010). ECWC is generally defined as an exaggerated cognitive and emotional affiliation with childhood and children (Finkelhor, 1984; Mann et al., 2010). An individual high in ECWC would be described as one whose emotional attachment and dependency needs are more easily met by interacting with children rather than with adults. They may seek child-oriented employment, report having children as friends, and report being in love with children.

ECWC predicts both the onset (e.g., McPhail et al., 2013; Konrad et al., 2018) and maintenance (Mann et al., 2010; McPhail et al., 2013) of sexual offending, and it is an important factor in many theories of sexual offending (e.g., Finkelhor, 1984; Seto, 2019; Ward & Beech, 2006). ECWC is related to risk of sexual recidivism, and may be important in the assessment, management, and treatment of individuals who commit sexual offenses (McPhail et al., 2013). Despite its importance in explaining sexual offending, the conceptualization of ECWC is not yet well understood. Several competing models are used to explain ECWC, but there is yet to be a consensus on which model best explains the development of ECWC. The current study tested various models of ECWC using a routine correctional sample of 983 men with a history of sexual offending against children (SOC) who were assessed for sexual recidivism risk in British Columbia (B.C.), Canada, between 2007 and 2013.

### Emotional Congruence with Children: what do We Know?

McPhail et al. (2013) conducted a meta-analysis of 30 unique samples to examine the prevalence of ECWC in individuals with a history of SOC compared to other groups of individuals without a history of SOC. They additionally examined ECWC’s relationship with treatment outcomes and recidivism. Individuals with a history of extrafamilial SOC evidenced higher levels of ECWC compared to those with histories of non-sexual offending, sexual offending against adults (SOA), and intrafamilial SOC. Individuals with a history of extrafamilial SOC with male victims evidenced higher levels of ECWC (*d*s ranging from 0.3 to 0.5) compared to those with histories of SOA, intrafamilial SOC, and extrafamilial SOC with female victims. ECWC was related to sexual recidivism among individuals with a history of extrafamilial SOC (*d* = 0.58, *n* = 639), but not among those with a history of intrafamilial SOC (*d* = −0.15, *n* = 893).

Integrating previous research and theory, McPhail et al. (2014) proposed three models of ECWC: the Blockage model, the Sexual Domain model, and the Psychological Immaturity model. The Blockage model predicts that ECWC is associated with a sense of loneliness and/or social rejection, a lack of stable intimate relationships with adult women, relatively low levels of sexual deviancy, compliance with authority figures, low impulsivity, and high hostility toward women. The Sexual Domain model predicts that ECWC is associated with sexual preoccupation, sexualized coping, sexual interest in children, cognitions supportive of adult-child sex, higher levels of pedophilic interests, and sexual offending against children that started young, persisted over time, and involved young victims, male victims, and many victims. Finally, the Psychological Immaturity model predicts that ECWC is associated with a younger offending age, developmental delay, poor cognitive-solving ability, low social competence, and high impulsivity.

McPhail et al. (2014) explored the correlates of ECWC as they relate to their proposed models in a sample of 221 men adjudicated for SOC. McPhail et al. (2014) used the “Emotional Identification with Children” item of the STABLE-2000 (Hanson et al., 2007) to classify participants as “high” or “low” on ECWC. This item is scored as a 0, 1, or 2, with higher scores indicating higher levels of ECWC. McPhail et al. (2014) categorized participants with a score of 0 as evidencing low ECWC and collapsed participants with scores of 1 or 2 as evidencing high ECWC. To examine the correlates of ECWC, McPhail et al. (2014) used area under the curve (AUC) analyses. The Sexual Domain model of ECWC was most supported in their sample, followed by the Blockage model, with the least amount of support for the Psychological Immaturity model.

Hermann et al. (2017) replicated McPhail et al.’s (2014) findings using a sample of 285 men adjudicated for SOC. Similar to McPhail et al. (2014), Hermann et al. (2017) dichotomized the ECWC item of the STABLE-2000. AUC analyses revealed that the Sexual Domain model was again most supported in their sample, with moderate support for the Blockage model of ECWC. Hermann et al. (2017), however, found additional differences between those high and low in ECWC that McPhail et al. (2014) either failed to detect or did not have enough data to examine. Hermann et al. (2017) uniquely observed that participants high in ECWC were more likely to have poor cognitive problem-solving skills, higher hostility toward women, more child victims, any unrelated victims, a male victim, lack of concern for others, and higher impulsivity compared to participants low in ECWC. Overall, however, both McPhail et al. (2014) and Hermann et al. (2017) found consistent associations between ECWC and atypical sexual interests, sexual preoccupation, and distorted cognitions about adult-child sex.

A potential fourth model, however, conceptualizes ECWC as an indicator of pedophilia (Brankley et al., 2019). In sexual interest, there are both physical and psychological aspects associated with that attraction (Martijn et al., 2020). ECWC has thus been conceptualized as the psychological aspect of attraction to children (Brankley, 2019). There is evidence to support this conceptualization; for example, in their analysis of the factor structure of the STABLE-2007, Etzler et al. (2020) found that the Emotional Identification with Children item of the STABLE-2007 loaded strongly onto their Sexual Deviance factor. Further, both McPhail et al. (2014) and Hermann et al. (2017) found the most support for their Sexual Domain model of ECWC. Both studies give evidence that ECWC is strongly related to atypical sexual interests and distorted cognitions about adult-child sex, which is likely capturing pedophilia. At the same time, however, the conceptualization of ECWC being the psychological aspect of pedophilia does not explain the strong relationship between sexual pre-occupation and ECWC that McPhail et al. (2014) and Hermann et al. (2017) both found. It is not clear, then, whether the conceptualization of ECWC being the psychological aspect of pedophilia is a part of the Sexual Domain model, or whether it is a separate, and more parsimonious explanation altogether.

McPhail et al. (2014) and Hermann et al. (2017) give important preliminary evidence for the models of ECWC; however, they have noteworthy limitations. Both McPhail et al. (2014) and Hermann et al. (2017) combined individuals with histories of SOC who had scores of 1 (‘some problem evident’) and 2 (‘significant problem evident’) on the Emotional Identification with Children items of the STABLE-2000/2007 into the high ECWC group; this grouping may miss important distinctions between those who scored a 1 versus a 2. The discrepant findings between these studies may be because their samples had varying numbers of those with scores of 1s and 2s in their samples. Additionally, both studies used samples of modest size (*N*_McPhail_ = 221, *N*_Hermann_ = 285).

Not all people who have a history of SOC are high in ECWC, and it is possible that those high in ECWC may follow different models. These distinctions are important to understand because they may give insight into how different subgroups of individuals with histories of SOC are differentially associated with ECWC. In other words, it is possible that instead of one model explaining ECWC, the models may reflect unique pathways by which subgroups end up developing ECWC. Each of these models has different proposed correlates, which implies that different treatment approaches may be necessary to reduce ECWC, and, subsequently, sexual recidivism, if these different subgroups exist.

### Current Study

To extend our understanding of the conceptualization of ECWC, we tested four hypothesized models of ECWC using a sample of 983 men with a history of SOC assessed for sexual recidivism risk in B.C. (a large Canadian province) between 2007 and 2013 (3 were hypothesized a prior based on McPhail et al., [2014]; the fourth was added as a post-hoc analysis). We extend the studies of McPhail et al. (2014) and Hermann et al. (2017) by incorporating Structural Equation Modelling (SEM), a rigorous approach to examining these models, and by using objective fit indices to determine which model best describes ECWC in our sample. Given that our sample is a routine correctional sample, the current study also provides a better estimate of the prevalence of each of these models (to the extent that they exist) than the previous studies. Additionally, we examined the men in our sample who are high, moderate, and low in ECWC separately, and did not collapse those with scores of 1 and 2 together in our analyses. With this more nuanced grouping, we can better determine if the correlates of ECWC are more endorsed by those most high in ECWC, compared to those with moderate and low ratings. As a post-hoc analysis, we also examined the hypothesis of ECWC being the psychological aspect of pedophilia, as proposed by Brankley (2019). To do so, we conducted a SEM for which only atypical sexual interests was entered as an indicator of ECWC.

McPhail et al.’s (2013) meta-analysis outlines the need for the relationship between ECWC and sexual recidivism to be examined using different subgroups of individuals who have a history of SOC. With Latent Class Analysis (LCA), we explored whether different subgroups of men with a history of SOC with ECWC emerge in our sample. Further, we examined the extent to which the characteristics of these subgroups (to the extent that they exist) mimicked the different models of ECWC. We additionally used Harrell’s *C* indices from Cox regression to assess whether ECWC was predictive of various forms of recidivism in each of the identified classes, and whether the subgroups identified by the LCA are differentially associated with recidivism. If subgroups of men with a history of SOC with ECWC exist, and if they are differentially associated with recidivism, this may inform individualized treatment approaches aimed at reducing ECWC.

## Method

### Participants

The current study was restricted to 983 men with a STABLE-2007 assessment who were released to the community (i.e., started a community supervision sentence or were released from jail or prison) prior to June 4, 2013, and who we could determine from administrative data had a sexual offense against a child somewhere in their sexual offense history. Additional exclusion criteria are discussed below, and the sample was further reduced to 377 men with ECWC (scored ≥1 on the STABLE ECWC item) for the Latent Class Analysis. Many individuals in this sample were scored on the STABLE-2007 multiple times; however, we included only the first assessment in the current study. Men who had only been scored on the STABLE-2000 were not included in our sample since the ECWC item of the STABLE-2000 is scored regardless of victim type, which made it impossible in our dataset to identify men with sexual offenses against children. In contrast, the STABLE-2007 ECWC item is only scored for individuals with sexual offenses against children.

On average, the justice-involved men in our sample were 42 years old (*SD* = 14.42). Self-reported race/ethnicity data (available for *n* = 955) indicated that the sample was predominantly White (64%; *n* = 607), followed by Indigenous (which includes First Nations, Métis, and Inuit, 21%; *n* = 199), East Asian (3%; *n* = 27), Indian (3%; *n* = 26), Hispanic (2%; *n* = 17), Black (2%; *n* = 15), and ‘other’ (7%; *n* = 65). Of those with self-reported data on education (*n* = 983), roughly half the sample (53.8%) had completed high school. On average, the sample was in Level III: Average Risk on Static-99R (*M* = 2.0, *SD* = 0.97, *n* = 966; Helmus et al., 2022) and Moderate Needs on the STABLE-2007 (*M* = 8.2, *SD* = 4.45, *n* = 983), with an average combined risk score of Level II: Below Average Risk *(M* = 1.9, *SD* = 0.84)*.* Most participants had no prior sexual offense charges (71%, *n* = 983; range 0–5).

### Exclusion Criteria

From our original sample (*N* = 4511), we removed participants with no “deviant” victims identified (as defined by the STABLE-2007 item definition; *n* = 1376), with only non-contact, non-solicitation charges (*n* = 90), or with missing data on the ECWC item of the STABLE-2007 (*n* = 1,964, this includes *n* = 1674 men scored on the STABLE-2000 version of the scale only [removed as we could not clearly identify if their victims were adults or children]). These exclusion criteria were implemented to help ensure that our sample consisted only of men who had at least one sex offense against a child. Due to the imperfect nature of administrative data from field settings (e.g., the possibility that some community supervision officers scored ECWC as ‘0’ for those without child victims as opposed to leaving it blank), it is possible that our sample definition may have included a small number of individuals whose sexual offending behaviors did not include child victims or missed some whose did. After removing participants with missing data on any of the indicator variables (*n* = 98), we were left with 983 men to be included in the SEM analyses.

The purpose of the LCA was to explore whether there were meaningful subgroups of individuals with ECWC. For these analyses, we removed participants who had a score of 0 (*n* = 704) on the Emotional Identification with Children item of the STABLE-2007; we only included participants who had a score of 1 (*n* = 327) or 2 on this item (*n* = 50). We used the same exclusion criteria in the Cox regression analyses and Harrell’s *C* estimates as in the LCA (*n* = 377). We conducted the Cox regression analyses and Harrell’s *C* estimates for each class identified by the LCA (see results; *n*_1_ = 132, *n*_2_ = 101, *n*_3_ = 144).

### Procedure

The original dataset included all individuals (*N* = 4511) supervised in the community by B.C. Corrections who received either a Static-99R, STABLE-2000, or STABLE-2007 assessment between January 1, 2005, and June 4, 2013. This sample would be considered a routine/complete sample under the definition used by Hanson et al. (2016). The current sample is from the field validity study described by Helmus et al. (2021). Informed consent was not obtained from participants because data were collected as part of policy for routine administration of correctional sentences. Consequently, this dataset would be considered a governmental administrative dataset, and government agencies are permitted to evaluate their practices for research purposes, either directly or indirectly, by sharing the data with researchers as part of their data sharing policies. This study is part of a larger programme of research using this dataset under a data sharing agreement and has received institutional ethics approval in Canada.

All risk assessments were scored by community supervision officers as part of routine case management practices. The educational background of the staff is unknown; however, the majority are presumed to have undergraduate degrees in a related field (i.e., psychology or criminology), although this is not necessarily true. While it would be uncommon for them to have advanced training in psychology or psychiatry, community supervision officers received certified training in scoring STABLE-2007 and underwent an annual peer-review process to ensure quality control. This is a field validity database, however, and there is no interrater reliability data for the assessments.

### Measures

#### STABLE-2007

The STABLE-2007 (Fernandez et al., 2014; Hanson et al., 2007) is an empirically derived risk scale assessing stable dynamic risk factors relevant to treatment and supervision of men convicted of a sexually motivated offense. It is the revised scale of the STABLE-2000 (see Hanson et al., 2007). This scale has 13 items, each scored as 0, 1, or 2 (reflecting no, some, and considerable concern). Total scores range from 0 to 26 for individuals with a victim under 14 years old, and 0 to 24 for others, because the Emotional Identification with Children item is only scored for the former group. Total scores were combined with Static-99R following the latest Evaluator Workbook (Brankley et al., 2019). The STABLE-2007 has acceptable field interrater reliability (ICC = .86; Fernandez & Helmus, 2017), including for the ECWC item (ICC = .81; 90% agreement). A meta-analysis found moderate accuracy of the STABLE-2007 in predicting sexual recidivism (AUC = .67), and it was incremental to Static-99R (Brankley et al., 2021). The STABLE-2007 has also demonstrated moderate predictive accuracy in field validity studies (Helmus et al., 2021). In the current sample, the STABLE-2007 and the ECWC item significantly predicted sexual recidivism (as per Helmus et al., 2021).

#### Recidivism

The dataset contains records of all charges and convictions within B.C. up to June 4, 2013 (see Helmus et al., 2021 for more details). Re-organizing the dataset into “sentencing occasion” clusters (see below) meant that pseudo-recidivism was not counted (i.e., new charges for old behavior). Offense details to determine sexual motivation were not available, so all classification decisions were based on the offense name in the charge/conviction. Offenses were classified into the following categories: non-contact sexual, contact sexual, non-sexual violent, non-violent, and technical (e.g., breach of probation); offense categorization followed the Static99 R coding rules (Phenix et al., 2016). From the categorizations above, this study analyzed four recidivism outcomes: any contact sexual recidivism, any sexual recidivism (contact or non-contact), any violent recidivism (which included contact sexual offenses but not non-contact), and any new crime recidivism (which included all offenses but excluded technical breaches).

Follow-up started after the conviction dates for those without custodial sentences or at release for those with custodial sentences. Of the men in our sample who had a score of at least 1 on the ECWC item of the STABLE-2007 (this was the sample included in recidivism analyses), the average length of their follow-up was 2.75 years (*SD* = 1.54, with a range of 0.11–5.55 years; *n* = 377). Recidivism rates were 26.3% (*n* = 99) for any recidivism, 11.1% (*n* = 42) for any violent recidivism, 5.3% (*n* = 20) for sexual recidivism, and 3.4% (*n* = 13) for contact sexual recidivism.

### Statistical Analysis

We report how we determined our sample size, all data exclusions, and all measures in the study. We also take responsibility for the integrity of the data (after it was provided to us by B.C. Corrections), the accuracy of the data analyses, and have made every effort to avoid inflating statistically significant results.

We conducted three sets of analyses. First, we conducted five separate SEMs, one for each of the proposed models of ECWC, one without specification, and a post-hoc model including only atypical sexual interests as an indicator variable. Absolute and comparative fit indices allowed us to determine which model best fit our data, and, therefore, which model best explained ECWC in our sample. We also used LCA to identify which subgroups of ECWC emerge in our data, and, specifically, if they resemble the three models of ECWC. Finally, we used Cox regression and Harrell’s *C* indices to determine which subtype of individuals with ECWC is most associated with recidivism. Each of these analyses is described below.

#### Structural Equation Modeling

**Figure 1.**
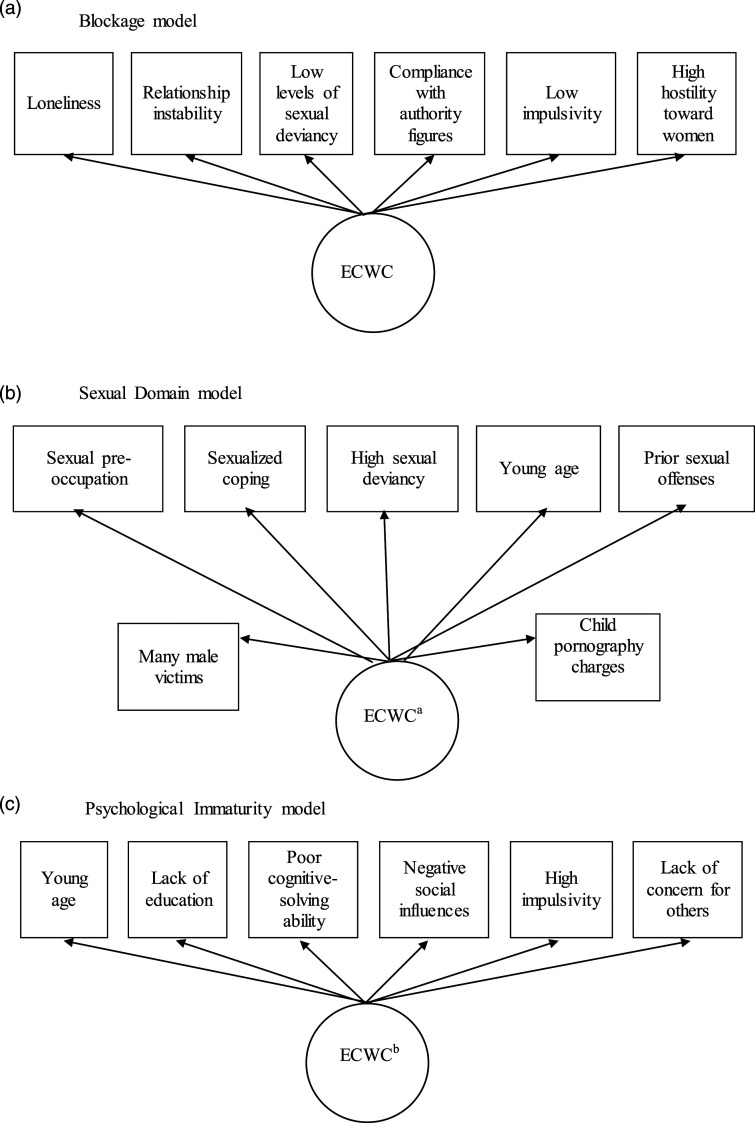
The three models of ECWC. Note. ^a^We are missing the following correlates, as outlined in the Sexual Domain model: cognitions supportive of adult-child sex, number of young victims, and total number of victims. We have included the Sexual Deviancy item of the STABLE-2007 as a proxy for sexual interest in children, prior sexual charges as a proxy for persistent sexual offending, and the combined age item of the Static-99 and Static-99R as a proxy for young age at first sexual offense. ^b^We have included education level as a proxy for developmental delay. We have included negative social influences and a lack of concern for others to account for low social competency.

To examine the three proposed models of ECWC, we conducted three separate SEMs, each based on one of the three models. Path diagrams illustrating these three models are presented in Figure 1. All SEM analyses were conducted using the lavaan package in R. For each model, we first allowed the parameters associated with each correlate of ECWC to be freely estimated by the models – this allowed us to see if the data contradicted the proposed empirical direction of the relationships. If these freely estimated parameters were associated with ECWC in the opposite direction of our hypothesized models, the models were re-specified and re-run with those parameters fixed in the predicted direction (the latter set of analyses are presented; see Part A of the Supplemental Materials for the freely estimated models). To determine which of these three models best fit our data, we compared each of them using the Vuong test, under the nonnest2 package in R. Supplemental Materials (Part A) describe the assumption testing.

To examine the adequacy of our model’s fit with our data, we used the following fit indices: the Comparative Fit Index (CFI), the Tucker-Lewis Index (TLI), the Root Mean Square Error of Approximation (RMSEA), and the Standardized Root Mean Squared Residual (SRMR). The CFI is an incremental measure of fit – values range from 0 to 1, with larger values indicating better model fit. A CFI of 0.90 or greater indicates acceptable model fit (Hu & Bentler, 1999). The TLI is also an incremental fit index, with no set range of values. Larger values indicate better model fit, with values greater than 0.90 (Byrne, 1994) or 0.95 (Schumacker & Lomax, 2004) indicating acceptable model fit. The RMSEA is an absolute measure of fit – values range from 0 to 1, with smaller values indicating better model fit. An RMSEA of 0.06 or less indicates acceptable model fit (Hu & Bentler, 1999). The SRMR is also an absolute measure of fit – values range from 0 to 1, with smaller values indicating better model fit. An SRMR of 0.08 or less indicates acceptable model fit (Hu & Bentler, 1999). If model fit is deemed acceptable, then parameter estimates can be examined. If unacceptable model fit is found, then the model can be revised (Kenny, 2020).

To compare the fit of the three models in predicting ECWC, we examined and compared the Akaike Information Criterions (AIC) and Bayesian Information Criterions (BIC) of each model according to Vuong’s theory (1989). The AIC and BIC are comparative measures of fit and are meaningful only when comparing them between models. A model is deemed to have better fit than another if its AIC and/or BIC values are comparatively smaller. We examined 95% confidence intervals of the differences in each models’ AIC and BIC values to determine whether these differences were statistically significant. To determine which of these three models best fit our data statistically, we compared each of them using Vuong’s test (1989).

With model comparison, it is not uncommon for absolute fit indices (e.g., RMSEA) to differ from comparative fit indices (e.g., BIC) in selecting the optimal model (Huang, 2017). When comparing two nested models (i.e., one model exists inside the other larger model), fit indices such as RMSEA and CFI are sufficient in comparing the two. With non-nested models, however, it is necessary to further compare them using the AIC and BIC, given that they include different manifest variables (Just Enough R. (n.d.)). When the decisions of these fit indices differ, it is up to the researcher to determine which comparison is more meaningful. AIC and BIC comparisons are more meaningful when selecting the model with the smallest population minimum discrepancy function, whereas an RMSEA comparison is more meaningful when selecting a model with the smallest population RMSEA (Huang, 2017). In our study, we are comparing multiple non-nested models, for which BIC has been shown to consistently select the most parsimonious one (Huang, 2017).

#### Latent Class Analysis

LCA was used to identify different subgroups within men who have a history of SOC and who evidence ECWC (i.e., a score of 1 or 2). Specifically, we used LCA to determine whether subgroups of men with a history of SOC and who have ECWC resemble the three models of ECWC and emerge as three separate subgroups. All correlates related to the three models of ECWC were included as indicator variables in the analysis. All LCAs were conducted using the poLCA package in R. Supplemental Materials (Part B) provides the information on statistical assumption testing.

The standard procedure for conducting an LCA is to first start with a one-class model, and to then add in classes one at a time to compare which model has the best fit. In selecting a final model of best fit, we examined multiple fit statistics. Specifically, between each model, we compared BIC and AIC values, for which smaller ICs indicate better fit. We additionally conducted Lo-Mendell-Rubin likelihood ratio tests (LMR-LRT) to determine whether adding an additional class to the model led to statistically better fit. As Nylund-Gibson and Choi (2018) discuss, sometimes fit indices and likelihood tests do not all point to the same number of classes. It is thus suggested to jointly consider the different fit statistics, interpretability and utility, and the classification diagnostics in determining the ideal number of classes to retain (Nylund-Gibson & Choi, 2018). We also considered parsimony in this process.

#### Cox Regression

Cox regression was used to examine whether the subgroups that emerged in the LCA were differentially associated with recidivism. Cox regression estimates hazard ratios from survival data. Hazard functions assess the risk that, at a particular moment in time, individuals will fail the outcome of interest if they have not already done so (in our case, the outcome being recidivism). To determine whether the individuals associated with each of the classes were differentially predictive of recidivism, we used class identification (i.e., 1, 2, or 3) as a grouping variable. We then examined whether the beta coefficients and hazard ratios associated with each pair of classes were significant.

**Harrell’s C.** Harrell’s *C* is a concordance index (effect size) that estimates the predictive accuracy of a model. The interpretation of Harrell’s *C* is the same as AUCs: an estimate of the probability that, of two randomly chosen individuals with a history of sexual offending, the one scoring higher risk on ECWC will reoffend before the other. Similar to AUCs, values of .56, .64, and .71 can be considered equivalent to Cohen’s day values of .20, .50, and .80, which are generally considered small, moderate, and large effect sizes (Helmus & Babchishin, 2017; Rice & Harris, 2005). Values close to 0.5 indicate no discrimination. We used the survival package in R to conduct the Cox regression and to obtain the Harrell’s *C* indices. Statistical assumptions are reported in Part C of the Supplemental Materials.

## Results

### Testing Model Fit: Structural Equation Modelling

The final three models of ECWC each showed relatively poor fit with our sample (see Table 1; correlates in the models are listed in Figure 1). We therefore ran an additional model with all predictors freely entered into the model to predict the latent variable of ECWC. This specification similarly produced a poorly fitted model. To examine the extent to which ECWC may reflect the psychological aspect of pedophilia, we conducted a post-hoc SEM for which only atypical sexual interests was inputted as an indicator variable. With only one indicator, we were unable to produce absolute fit indices for this model. Consequently, we examined the bivariate relationship between ECWC and atypical sexual interests and found a moderate relationship (Kendall’s tau = .367, 95% CI of .343–.391.).

**Table 1.** Model Fit for Three Models of ECWC.

Model of ECWC	Fit Indices			
	RMSEA	SRMR	CFI	TLI
Blockage	0.238	0.224	0.101	−0.498
Sexual domain	0.098	0.069	0.740	0.610
Psychological immaturity	0.105	0.066	0.856	0.784
Freely estimated model	0.096	0.080	0.664	0.612

*Note.* RMSEA = Root mean square error of approximation; SRMR = Standardized root mean squared residual; CFI = Comparative fit index; TLI = Tucker-Lewis index. An RMSEA of 0.06 or less indicates acceptable model fit (Hu & Bentler, 1999). An SRMR of 0.08 or less indicates acceptable model fit (Hu & Bentler, 1999). A CFI of 0.90 or greater indicates acceptable model fit (Hu & Bentler, 1999). A TLI values greater than 0.90 (Byrne, 1994) or 0.95 (Schumacker & Lomax, 2004) indicating acceptable model fit.

Model comparisons are presented in Table 2. All three models of ECWC showed significantly better fit than the freely estimated model (*p* < .001). The Sexual Domain model had comparatively better fit than the other three models of ECWC (all *p*s < .001; see Table 2). Within the models, the Sexual Domain model showed significantly better fit (*p* < .001) compared to the Psychological Immaturity model. Although Table 1 shows that the Blockage model had The worst absolute fit indices, according to Vuong’s test (1989), the Blockage model showed significantly better comparative fit (*p* < .001) with our sample than the Sexual Domain and Psychological Immaturity models.

**Table 2.** SEM Model Comparisons.

Model Comparisons	AIC	BIC	95% CI [LL, UL]	
			AIC_1_ – AIC_2_	BIC_1_ – BIC_2_	*z* ^a^
Blockage (baseline)	1109.39	11,032.70			
Sexual domain			**[**−**720.40, 10.59]**	[−730.18, 0.81]	1.88*
Psych. Immaturity			[−5482.01, −4985.43]	[−5491.79, −4995.21]	41.28**
Full			[−18510.13, −17695.50]	[−18607.94, −17793.31]	86.92**
Sexual domain			[9537.24, 9948.43]	[9591.04, 10002.22]	92.67**^b^
Sexual domain (baseline)	11387.61	11,456.08			
Psych. Immaturity			[−5261.36, −4496.27]	[−5261.36, −4496.27]	25.00**
Full			[−18058.13, −17437.68]	[−18146.16, −17525.71]	111.90**
Sexual domain			[9761.03, 10434.44]	[9824.61, 10498.02]	58.63**^b^
Psych. Immaturity (baseline)	16266.42	16,334.89			
Full			[−13285.30, −12452.89]	[−13373.33, −12540.92]	60.43**
Sexual domain			[14758.22, 15194.88]	[14821.80, 15258.46]	134.21**^b^
Full (baseline)	29137.32	29,293.82			
Sexual domain			[27369.29, 28322.00]	[27520.90, 28473.61]	114.32**^b^
Sexual domain	1289.87	1294.76			

*Note.* Bolded values indicate non-significant CIs.

^b^Model 2 has better fit than Model 1.

^a^Vuong test results.

*p < .05. **p < .001.

The worst absolute fit indices, according to Vuong’s test (1989), the Blockage model showed significantly better comparative fit (*p* < .001) with our sample than the Sexual Domain and Psychological Immaturity models.

### Latent Class Analysis

Based on our sample and included indicator variables, LCA identifies the number of classes that are present, and the probability of each indicator being associated with a particular class. A three-class solution emerged as best fitting with our data^1^, indicating three subgroups of men with a history of SOC who demonstrate ECWC in our sample. In Table 3, we present the pattern of factor loadings for the three classes. These three subgroups generally did not mimic the three models of ECWC.

**Table 3.** A Three-Class Solution of Men with Histories of SOC who are High in ECWC.

	Class 1 (*n* = 132)		Class 2 (*n* = 101)		Class 3 (*n* = 144)	
Predictor	Low	High	Low	High	Low	High
Loneliness	.48	.52	.23	**.77**	.08	**.92**
Relationship instability	.34	**.66**	.07	**.93**	.09	**.91**
Sexual deviancy	.41	.59	1	0	.40	**.60**
Lack of cooperation with supervision	**.84**	.16	.77	.23	.43	.57
Impulsivity	**.82**	.18	.47	.53	.15	**.85**
Hostility toward women	**.81**	.19	.72	.28	.42	.58
Sexual preoccupation	.59	.41	.68	.32	.15	**.85**
Sexualized coping	**.66**	.34	.68	.32	.29	**.71**
Young age	**.68**	.32	.28	**.72**	.40	**.60**
Prior sexual charges	**.73**	.27	.87	.13	.54	.46
Prior or index CP charges	**.86**	.14	.96	.04	**.85**	.15
Any male victims	**.72**	.28	.98	.02	**.75**	.25
Poor cognitive problem solving	.57	.43	.15	**.85**	.04	**.96**
Negative social influence	**.62**	.38	.52	.48	.28	**.72**
Lack of concern for others	**.76**	.24	.73	.27	.24	**.76**
Lack of high school completion	**.67**	.33	.36	**.64**	.44	.56

*Note.* Class 1: Relationship Deficits (low risk); Class 2: Immature/Lonely; Class 3: High Risk. Bolded values indicate an association with either the high or low endorsement of each specific item. If neither high nor low are bolded for a predictor, this indicates that that predictor is not present in that specific class. CP = Child pornography.

The first class (35% of the sample, *n* = 132) was characterized by older men who lacked relationship stability, who completed high school, and who were low on the indicators of sexual deviancy and sexual self-regulation problems, as well as on the indicators of general criminality. We have labelled this as the “Relationship Deficits (low risk)” class. The second class (27% of the sample, *n* = 101) was characterized by younger men who have problems with self-regulation and cognitive problem solving, and who did not complete high school. These men additionally scored low on atypical sexual interest and the sexual-self regulation indicators, as well as the indicators of general criminality. We have labelled this class as the “Immature/Lonely” class. The third class (38% of the sample, *n* = 144) was characterized by young men who have problems with self-regulation and cognitive problem solving, and who score high on sexual deviancy and the sexual self-regulation indicators, as well as the indicators of general criminality. We have labelled this class as the “High Risk” class. We were surprised that having a male victim did not load onto any of these classes given its previous association with ECWC (McPhail et al., 2013). In a post-hoc analysis we observed a small positive relationship between having a male victim and ECWC in the full sample (*r*_pb_ = .14, *p* < .001, for all *N* = 983 men included in our SEM analyses, AUC = .58, 95% CI [.53, .64], *N* = 983).

### Cox Regression

In Table 4, we present the recidivism rates associated with each class. Class 3 – High Risk – had the highest recidivism rate with a 2-year recidivism rate for any recidivism of 50.5% compared to 16.3% for Class 1 – Relationship Deficits (low risk).

**Table 4.** 2-Year Recidivism Rates by Class Association.

	Class 1 (*n* = 86)		Class 2 (*n* = 60)		Class 3 (*n* = 95)	
Recidivism type	*N*_*rec*_	%	*N*_*rec*_	%	*N*_*rec*_	%
Any sexual	3	3.49	4	6.67	11	11.58
Sexual contact	2	2.32	4	6.67	7	7.37
Violent (including sexual)	9	10.46	9	15.00	22	23.16
Any	14	16.28	23	38.33	48	50.53

*Note.* Class 1: Relationship Deficits (low risk); Class 2: Immature/Lonely; Class 3: High Risk.

In Table 5, we present the results of our Cox regression analyses using class association as a grouping variable to examine differences in recidivism rates. Classes 1 and 2, the Relationship Deficits (low risk) and Lonely/Immature classes, differed significantly in any recidivism, with Class 2 being more likely to re-offend compared to Class 1. Classes 1 and 3, the Relationship Deficits and High Risk classes, differed significantly in their risk for sexual, violent, and any recidivism, with Class 3 being consistently more likely to re-offend compared to Class 1. Classes 2 and 3, the Lonely/Immature and High Risk classes, did not differ significantly in their risk for any type of recidivism.

**Table 5.** Cox Regression comparing Recidivism Rates across LCA Classes.

Recidivism	Classes^a^	*B*	HR	*p*	*C*	95% CI	
						Lower	Upper
Any sexual					.57	.41	.72
	1 versus 2	0.74	2.09	.335			
	1 versus 3	1.55	4.70	.016*			
	2 versus 3	0.81	2.25	.158			
Sexual contact					.50	.31	.68
	1 versus 2	1.08	2.96	.211			
	1 versus 3	1.24	3.46	.122			
	2 versus 3	0.16	1.17	.802			
Violent (including sexual)					.54	.44	.64
	1 versus 2	0.51	1.66	.271			
	1 versus 3	0.96	2.62	.014*			
	2 versus 3	0.46	1.58	.228			
Any					.55	.49	.61
	1 versus 2	1.10	3.01	<.001**			
	1 versus 3	1.45	4.26	<.001**			
	2 versus 3	0.35	1.42	.133			

*Note.* Class 1: Relationship Deficits (low risk); Class 2: Immature/Lonely; Class 3: High Risk.

^a^*n*_1_ = 132; *n*_2_ = 101; *n*_3_ = 144.

*p < .05. **p < .001.

In Table 6, we present the predictive accuracy of ECWC (as indexed by Harrell’s *C*) for each class. All participants assigned to classes had at least a score of 1 on ECWC, so this analysis tested if those scoring 2 on ECWC have higher recidivism rates than those scoring 1 on ECWC. Generally, higher score on ECWC were associated with higher risk for reoffending, especially for sexual contact recidivism. Non-overlapping 84% Harrell’s *C* CIs indicate that the predictive accuracy of ECWC is not statistically different across classes (at *p* < .05 level). All classes overlapped on their 84% CIs, indicating that there were no differential relationships between ECWC and recidivism for the three classes. In other words, the association between ECWC and recidivism were similar across the three classes.

**Table 6.** The Predictive Accuracy of ECWC for Three Classes.

	Class^a^	Harrell’s *C*	95% CI		84% CI	
Recidivism type			Lower	Upper	Lower	Upper
Any sexual^b^	1	.54	[.34	.73]	[.40	.68]
	2	**.54**	[.51	.56]	[.52	.56]
	3	**.61**	[.46	.76]	[.50	.72]
Sexual contact^c^	1	**.56**	[.53	.60]	[.54	.59]
	2	**.53**	[.51	.56]	[.52	.56]
	3	**.65**	[.45	.84]	[.50	.79]
Violent (including Sexual)^d^	1	.49	[.36	.62]	[.39	.59]
	2	**.54**	[.51	.57]	[.52	.56]
	3	.50	[.41	.59]	[.43	.56]
Any^e^	1	.53	[.43	.64]	[.46	.61]
	2	.52	[.48	.56]	[.49	.55]
	3	.52	[.47	.58]	[.48	.56]

*Note.* Class 1: Relationship Deficits (low risk); Class 2: Immature/Lonely; Class 3: High Risk. CI = Confidence interval. Bolded values indicate significance. Non-overlapping 84% CI indicates that the predictive accuracy of ECWC is not statistically different across classes (*p* < .05).

^a^*n*_1_ = 132; *n*_2_ = 101; *n*_3_ = 144.

^b^Average follow-up time in years: *m*_1_ = 2.87 (1.57), *m*_2_ = 2.49 (1.56), *m*_3_ = 2.62 (1.51).

^c^Average follow-up time in years: *m*_1_ = 2.88 (1.58), *m*_2_ = 2.49 (1.56), *m*_3_ = 2.64 (1.50).

^d^Average follow-up time in years: *m*_1_ = 2.76 (1.61), *m*_2_ = 2.32 (1.59), *m*_3_ = 2.36 (1.47).

^e^Average follow-up time in years: *m*_1_ = 2.60 (1.62), *m*_2_ = 1.91 (1.63), *m*_3_ = 1.80 (1.51).

## Discussion

Using a routine correctional sample of 983 men on community supervision in British Columbia (B.C.) between 2007 to 2013 with a history of sexually offending against children (SOC), we investigated the extent to which various models of emotional congruence with children (ECWC) are empirically supported. To do so, we used structural equation modelling (SEM) to test the fit of various models in explaining ECWC in our sample. Overall, our results indicated that the Psychological Immaturity model provided the worst fit in explaining ECWC. The Blockage model provided the best comparative fit of the three McPhail et al. (2014) models, although it was not significantly better than the Sexual Domain model in explaining ECWC^2^. These three models all had relatively poor absolute fit indices, suggesting that these models did not explain ECWC well in this routine correctional sample. Compared to McPhail et al.’s (2014) three models of ECWC, our post-hoc model with only atypical sexual interests as an indicator of ECWC best fit our data (we could not generate absolute fit indices for a single variable, but the bivariate relationship with ECWC was moderate). This supports Brankley’s (2019) conceptualization of ECWC as the psychological aspect of pedophilic interest, rather than a distinctly unique construct (as conceptualized by McPhail et al., 2014).

The exploratory model, for which we included all the predictor variables associated with the three models of ECWC, had the worst fit (both absolute and comparative). This may be unsurprising, given that SEM should be theoretically driven, and inputting all variables into one model is atheoretical, whereas the other models of ECWC were each theoretically informed. If anything, however, the finding that this exploratory model had the poorest fit compared to the proposed ones shows the models’ relative incremental fit in explaining ECWC and may provide (weak) support for them.

The Psychological Immaturity model had the poorest fit of the three models of ECWC. This may not be surprising given that ECWC is more specific to sexual offending than general offending, and correlates associated with this model are all indicators of general criminality. Indicators of general criminality are assumed to be positively linked with ECWC in this model. The literature on general criminality, however, shows that general criminality is negatively correlated with sexual criminality, and specifically with ECWC and pedophilia (Brouillette-Alarie & Proulx, 2019; Brouillette-Alarie & Hanson, 2015; Brouillette-Alarie et al., 2018). In exploring the taxonomy of pedophilia, Brankley (2019) found that individuals identified by the pedophilia taxon were low on general criminality as defined by Brouillette-Alarie and Hanson (2015), and yet they were high on ECWC. If the relationship between general criminality and ECWC is negative, then this would explain why the Psychological Immaturity model provided such poor fit in explaining ECWC and provides support for the negative relationship between general criminality and ECWC observed in previous research.

We performed a latent class analysis (LCA) to explore whether there are subgroups of men with a history of SOC who have ECWC. We included all the indicator variables from our SEM analyses in the LCA. Three classes were identified as best fitting the data, although none of these classes directly mimicked the three models of ECWC. The first class, which we have named the “Relationship Deficits (low risk)” class, included men with a history of SOC with ECWC who lack relationship stability, and were characterized by low sexual and general criminality. This class may be a weak manifestation of the Blockage model of ECWC. Most specifically, this class is defined by a lack of relationship history. It may be that the ECWC experienced by these men is because of some interpersonal deficit blocking them from establishing adult relationships. The second class, which we have named the “Immature/Lonely” class, included men who were young and showed problems with self-regulation and cognitive problem-solving, characterized by low sexual and general criminality. This class does seem to be related to the Psychological Immaturity model of ECWC; however, only on a purely immature level. This may suggest that the ECWC experienced by the men in this class is because of pure immaturity and loneliness, and not driven by an underlying characteristic of criminality, as proposed by McPhail et al. (2014). The last class, which we have named the “High Risk” class, included men who were also young and showing problems with self-regulation and cognitive problem-solving, but who were characterized by high sexual and general criminality. This class may reflect the Sexual Domain model of ECWC, and/or it may encapsulate men who experience ECWC as the psychological aspect of pedophilia. In combination with our SEM results, our results do not fully support or refute McPhail et al.’s (2014) models of ECWC; however, they may indicate that these models are incomplete or insufficient in explaining it.

Interestingly, having a male victim did not load onto any of the classes. This was contrary to our hypothesis given that men with a history of SOC who have extrafamilial male victims typically score higher on ECWC (McPhail et al., 2013). We may not have observed this relationship because our participants were drawn from a routine correctional sample, rather than a treatment or higher risk sample; however, we still had a substantial portion of men with a history of SOC with ECWC in our sample who had male victims (*n* = 76, 20%). If ECWC does simply represent the psychological attraction aspect of pedophilia (Brankley, 2019; Martijn et al., 2020), then this would explain why the association between having at least one male victim and ECWC is small, although present, but did not load onto any of the classes in this study.

We lastly examined whether ECWC was differentially associated with recidivism across the three classes, as well as whether the three classes were differentially associated with recidivism. ECWC predicted recidivism similarly across the three classes; however, the men associated with these classes are different in their likelihood of some types of recidivism. Given how each of these classes are characterized, our results are perhaps unsurprising. The men in Class 3 (High risk) were consistently more likely to re-offend with sexual, violent, and any crimes compared to those in Class 1 (Low risk). Of the men with low levels of sexual and general criminality, those with high self-regulation issues (Class 2) were more likely to have any re-offense compared to those without self-regulation issues (Class 1). This pattern of results indicates that self-regulation may play a key role in recidivism risk for men with a history of SOC with ECWC who are otherwise generally low in sexual and general criminality. In fact, even when characterized by low risk, self-regulation issues may increase risk of re-offending to the levels seen by men in Class 3, characterized by high risk.

### Strengths and Limitations

We used an administrative dataset collected by B.C. corrections of 983 men with sexual offenses against children between 2007 and 2013. We also used more objective tests of model fit compared to McPhail et al. (2014) and Hermann et al. (2017), who each examined the likelihood of the correlates of ECWC being more endorsed by individuals with a history of SOC who are high in ECWC compared to those who are not, rather than examining the model fit. SEM allowed us to use objective statistical tests to directly examine the fit of these three models in explaining ECWC. We were also able to distinguish between ECWC scores of 1 and 2, whereas the two previous studies grouped them together. We incorporated a much larger sample in our study than McPhail et al. (2014) and Hermann et al. (2017) combined, allowing greater statistical power and more fine-tuned analyses. In our larger sample, we additionally had more individuals with scores of 1 and 2 on the Emotional Identification with Children item of the STABLE-2007, which may have given us a more representative sample of men with a history of SOC who are each high, moderate, and low in ECWC. The benefit of our sample is that it provided us with a representative cohort of justice-involved individuals with a known history of SOC. The limitation of this sample is that our exploration is restricted to what is collected by the agency.

Overall, we did not find a clear winning model explaining ECWC in our sample. We may have found poor fit because we were missing key indicators of ECWC that can explain it better than the ones we included in our SEM analyses. It could also be that the indicators that we included were insufficient; most indicator variables were assessed by only one risk scale item scored as 0, 1, or 2. The ECWC item of the STABLE-2007 was scored in routine practice based on interview and file reviews; however, this may not be an ideal assessment of ECWC. Instead, a self-report measure may be a more reliable and valid assessment of ECWC. Unfortunately, large correctional datasets are typically restricted to short and easy to score measures, so although a strength of our study is the inclusion of a large and representative sample, it is also the cause of these limitations.

Our sample size was also relatively small for our LCA analyses (*n* = 377), and, as such, it would be important to replicate the findings in an independent sample to determine the veracity of the three-class solution. In our sample, we also did not know the relationship of the victim(s) to these men, so we were unable to distinguish between intrafamilial and extrafamilial SOC. This is an important limitation, given that meta-analyses have found differences in ECWC levels between these two groups (McPhail et al., 2013; Seto et al., 2016). Our dataset additionally did not specify victim age, and so we had to use a proxy variable for contact SOC. In theory, the ECWC item of the STABLE-2007 should only be scored for individuals who have at least one victim under the age of 14 years. Given this specification, we excluded any men from our dataset who were not scored on this or who were scored as not having a “deviant” sexual offense victim on the STABLE-2007 (given that children are subsumed in the deviant victim item). It is nonetheless possible that our sample included men with an exclusive history of SOA.

Finally, the men included in this dataset were assessed multiple times. In our analyses, we included only the first STABLE-2007 assessment; however, these men were actively undertaking community supervision during the follow-up. Although we do not know whether they attended any programming, studies have found that community supervision can reduce risk scores and recidivism (Babchishin & Hanson, 2020; Bonta et al., 2021; Lloyd et al., 2020). In addition, it has been shown that treatment can significantly reduce levels of ECWC (McPhail et al., 2013). As such, the relationship between recidivism and ECWC variables may be confounded by changes in ECWC that are not captured in our study. Similarly, the recidivism follow-up period in our sample was relatively short (2.8 years). It is therefore unclear whether the relationship that we have observed would extend to long-term recidivism risk.

### Practical and Theoretical Implications

The current study suggests that the Blockage and Sexual Domain models of ECWC may be useful, albeit limited, conceptualizations of ECWC and found the strongest support for ECWC being an indicator of pedophilia rather than a distinct construct. Programming that targets ECWC and the indicators identified by the Blockage model – loneliness, relationship instability, and hostility toward women – and the Sexual Domain model – sexual preoccupation, sexualized coping, and cognitions supportive of adult-child sex – may contribute to a reduction in ECWC. Conversely, programming that targets ECWC and the indicators of general criminality that are identified by the Psychological Immaturity model are unlikely to influence ECWC.

We found that the model including only atypical sexual interests as an indicator of ECWC fit our data significantly better than either the Blockage or Sexual Domain models of ECWC. This may give support to the conceptualization of ECWC being the psychological aspect of sexual interest in children (Brankley, 2019; Martijn et al., 2020). This would indicate, then, that men with pedophilic interests should also exhibit high levels of ECWC. That said, our study suggests it is not that simple. Only one of the classes identified by our LCA fit the conceptualization of ECWC as an indicator of pedophilia. In our LCA, we found that atypical sexual interests were unrelated to Class 1, and members of Class 2 were identified as having low levels of atypical sexual interests. Only the men in Class 3 were characterized by high levels of atypical sexual interests. These results therefore suggest that ECWC is likely a multifaceted construct, and individuals may exhibit high levels of ECWC for various reasons.

As discussed, our LCA defined the men in Class 1 as experiencing high levels of relationship deficits, which may indicate that these men are blocked from healthy relationships with adults, as outlined in the Blockage model of ECWC. As a result of this blockage, these men may seek out the company of children to fulfill their relationship needs, given that children may be easier targets. In this case, blockage from healthy relationships may explain the high levels of ECWC experienced by these men, despite having relatively low levels of atypical sexual interests. Similarly, the men in Class 2 may seek out the company of children as a direct result of their loneliness and immaturity, which define this class. This, then, might explain the high levels of ECWC experienced by these men, while again having relatively low levels of atypical sexual interests. On the other hand, individuals may also exhibit high levels of ECWC because of their sexual interest in children. Specifically, their high levels of ECWC may be due to the psychological attraction that they feel towards children. In this circumstance, these individuals would exhibit high levels of both ECWC and atypical sexual interests. This is most clearly reflected in the men defined by Class 3 of our LCA.

Our findings suggest that ECWC is complex and that there are various paths towards ECWC. These paths are likely meaningful for treatment. While our results suggest that ECWC is not differentially associated with recidivism across the three classes, they do suggest that the three classes are differentially associated with recidivism. The men in Class 1 were much less likely to reoffend compared to the men in both Classes 2 and 3, who showed no differences in their recidivism risk. The factors associated with each of the three classes likely reflect the treatment needs of the men associated with each of them. For example, the men identified by Classes 1 and 2 are unlikely to benefit from a treatment program targeting management of atypical sexual interests, whereas the men identified by Class 3 likely would.

Taken together, our results suggest that there are different pathways for which ECWC may be expressed, and that not all these paths are explained by the individuals’ atypical sexual interests. Consideration of both an individual’s ECWC subgroup association, in addition to the reason underlying their ECWC, may be necessary in informing effective individualized treatment programs aimed at reducing ECWC.

### Future Research Directions

Future research is needed to explore whether men with histories of SOC with ECWC respond differently to treatment aimed at reducing ECWC depending on their ECWC subgroup association and the underlying explanation for their ECWC. We suggest that the indicators associated with the Blockage and Sexual Domain models and the variables characterized by each of the classes of ECWC may be useful as treatment targets in programming, but future research is needed to explore their effectiveness in reducing ECWC. To assess this, men with histories of SOC who are high in ECWC should be followed and continuously assessed throughout treatment programs that specifically implement these correlates as treatment targets. If ECWC levels are reduced following the treatment program, this may lend support to targeting these correlates in treatment programs. Further, if treatment targets are differentiated by ECWC class association, the effectiveness of programming in reducing ECWC in each of these classes will inform the importance of their consideration for programming.

Finally, the results of our study should be replicated in future research. While we have given limited support to McPhail et al.’s (2014) three models of ECWC and Brankley’s (2019) conceptualization of ECWC as an indicator of pedophilia, we have noteworthy limitations that may account for this. Future research should incorporate better measurements of ECWC and pedophilia, with a larger number of men with a history of SOC.

### Conclusion

Overall, we found limited support for McPhail et al.’s (2014) three models of ECWC and, instead, found that ECWC was best explained as an indicator of pedophilia. That said, not all men with ECWC in our sample had pedophilia. Instead, we found three subgroups of men with ECWC: the Relationship Deficits (low risk), Immature/Lonely, and High-Risk classes of ECWC. These findings highlight that the conceptualization of ECWC is still incomplete and stress the fundamental need for accurate measurement that prioritizes construct validity over predictive ability.

## Supplemental Material

Supplemental Material - Emotional Congruence with Children: An Empirical Examination of Different Models in Men with a History of Sexually Offending Against Children Supplemental Material for Emotional Congruence with Children: An Empirical Examination of Different Models in Men with a History of Sexually Offending Against Children by Julia M. Fraser, Kelly M. Babchishin, and L. Maaike Helmus in Sexual Abuse

## Notes

1. Compared to the two-class solution, the three-class solution had a slightly higher BIC value, but a substantially smaller AIC value. Additionally, the LMR-LRT indicated that the three-class solution had statistically better fit than the two-class solution. Compared to the three-class solution, the four-class solution had a higher BIC, and a slightly smaller AIC. The LMR-LRT also indicated that the four-class solution had statistically better fit than the three-class solution. We generated elbow plots to examine where the BIC and AIC values leveled out to determine the accurate number of classes to retain. Between the models with two and three classes, there was a very small BIC value change, with each subsequent BIC increase being larger. Between the models with two and three classes, there was a large AIC value change, with each subsequent AIC decrease being smaller. Both elbow plots indicated that the BIC and AIC values level out at the three-class solution. The three-class solution additionally has more substantive interpretability and utility than the two-class and four-class solution; therefore, we have retained the three-class solution. (See Part B of the Supplement Materials for additional details).

2. A model’s absolute fit reflects the extent to which it fits the data, whereas comparative fit indices reflect which model, among two or more, better fits the data. It is not uncommon for absolute and comparative fit indices to point to different models as best fitting the data. We found that the Blockage model provided the worst absolute fit indices relative to the Sexual Domain and Psychological Immaturity models (Table 1), but it had comparatively better fit than the other two. It is perhaps worth noting that, prior to our re-specification of the Blockage model for which we forced all of the predictor variables to be in the predicted direction according to this model, the un-specified version of this model showed relatively good model fit and demonstrated both better absolute and comparative fit than the other models (see Part A of the Supplemental Materials). This may explain why, comparatively, the Blockage model showed better fit than the others, regardless of the absolute fit indices associated with this model’s re-specification.
