# Supplementary material for: Emotional Congruence with Children: An Empirical Examination of Different Models in Men with a History of Sexually Offending Against Children
Source: Sex Abuse. 2023 Jun 5;36(5):546–71. doi: 10.1177/10790632231172160 (PMC11177559; doi:10.1177/10790632231172160)
Supplement: Supplemental Material - Emotional Congruence with Children: An Empirical Examination of Different Models in Men with a History of Sexually Offending Against Children [file sj-pdf-1-sax-10.1177_10790632231172160.pdf]

Emotional Congruence with Children: An Empirical Examination of Different Models in Men with a History of Sexually Offending Against Children

Supplemental Online Materials

Part A: SEM

We used structural equation modelling (SEM) analyses to determine which model of emotional congruence with children (ECWC) would have the best fit with our sample. We conducted three separate SEMs, one for each model of ECWC. In each model, the predictor (measured) variables were those correlates of ECWC associated with each separate model, with ECWC as the latent (unmeasured) variable (see Table 1). All SEM analyses were conducted using the lavaan package in R (all syntax are provided in the Appendix of this document).

Table 1

The Correlates of ECWC Associated with Three Models of ECWC.

	Models of ECWC		
Correlates of ECWC	Blockage	Sexual Domain	Psychological Immaturity
Loneliness	High		
Relationship instability	High		
Sexual deviancy	Low		
Lack of compliance with authority figures	High		
Impulsivity	Low		High
Hostility toward women	High		
Sexual preoccupation		High	
Sexualized coping		High	
Young age		Low	Low
Prior sexual charges		High	
Prior or index child pornography offenses		High	
Number of male victims		High	
Difficulty with problem solving			High
Negative social influences			High
Lack of concern			High
Education level			Low

Note. High/low labels according to the predicted of each correlate relationship with ECWC under each model.

Assumptions

SEM is impacted by sample size, multivariate normality, outliers, and missing data. To ensure that it is appropriate to conduct SEM analyses with our data, a ratio of 5:1 between number of participants and number of parameters is required; however, a 10:1 or 20:1 ratio is preferable. For the Blockage model, we have a ratio of 983:12; for the Sexual Domain model, we have a ratio of 983:18; for the Psychological Immaturity model, we have a ratio of 983:16. When run in the Sexual Domain model, the age variable had a variance that was 200 times that of the other variables in the model. We therefore rescaled this variable by multiplying it by 0.1 to ensure accurate model fit with all of our parameters (Hallquist, 2018). The re-scaled age variable was used in place of the raw age variable for all SEM analyses.

Based on the reviewed literature, we have specified three separate models to examine using SEM. In the Blockage model, we have 6 variables in our matrix and 21 elements in our initial covariance matrix. We have 6 observed variable variances and 6 factor loadings; thus, we have 12 free parameters to estimate in this model. Our degrees of freedom (df) for this model are 9, which indicates that all of our free parameters can be estimated, and our model is over identified. In the Sexual Domain model, we have 9 variables in our matrix, and 45 elements in our initial covariance matrix. We have 9 observed variable variances and 9 factor loadings; thus, we have 18 free parameters to estimate in this model. Our df for this model is 27, which indicates that all of our free parameters can be estimated, and our model is over identified. In the Psychological Immaturity model, we have 8 variables in our matrix, and 36 elements in our initial covariance matrix. We have 8 observed variable variances and 8 factor loadings; thus, we have 16 free parameters to estimate in this model. Our df for this model is 20, which indicates that all of our free parameters can be estimated, and our model is over identified.

The Blockage Model

Assumptions

We assessed for multivariate normality using the E-statistic Energy test, by examining multivariate skewness and kurtosis values, and by examining Q-Q plots for each variable. The E-statistic and skewness and kurtosis values were all non-significant, indicating multivariate normality. Many of our variables are coded categorically, according to the STABLE-2007, so we examined Q-Q plots based on generated distributions according to each variable's sample size, mean, and standard deviation. All Q-Q plots followed normal distributions. We assessed for outliers using the Grubbs test, which revealed no high or low outliers for any of the variables.

Model Fit

To examine the fit of the Blockage Model, we entered the variables associated with loneliness, a lack of a stable intimate relationship with women, sexual deviancy, compliance with authority figures, impulsivity, and hostility, to predict the latent variable of ECWC. We first freely entered these variables, allowing the model to estimate the values of each parameter. With this model specification, the model had adequate model fit (RMSEA of 0.066, SRMR of 0.035, CFI of 0.931, and a TLI of 0.886). Not all parameters, however, were being predicted accurately according to the Blockage Model. Specifically, sexual deviancy and impulsivity were predicted to be positively associated with ECWC, whereas under the Blockage Model, sexual deviancy and impulsivity are hypothesized as being negatively associated with ECWC.

To examine the fit of the Blockage Model more accurately with our data, we fixed the parameters of sexual deviancy and impulsivity to be negative. This new re-specification led to poor model fit (see Table 2).

Table 2

Model Fit of Three Models of ECWC

	Fit Indices			
Model of ECWC	RMSEA	SRMR	CFI[a]	TLI
Blockage	0.238	0.224	0.101	-0.498
Sexual Domain	0.196	0.245	0.318	0.091
Psychological Immaturity	0.118	0.079	0.771	0.679
Freely estimated model	0.185	0.134	0.261	0.179

Note. RMSEA = Root mean square error of approximation; SRMR = Standardized root mean squared residual; CFI = Comparative fit index; TLI = Tucker-Lewis index.

The Sexual Domain Model

Assumptions

To assess for multivariate normality, we again used the E-statistic Energy test, examined the multivariate skewness and kurtosis values, and examined Q-Q plots for each variable. The E-statistic and skewness and kurtosis values were all non-significant, indicating multivariate normality. We again examined Q-Q plots based on generated distributions according to each variable's sample size, mean, and standard deviation, which all followed normal distributions. We assessed for outliers using the Grubbs test, which revealed a high outlier for the prior sex charges variable. The highest value was a 5, with the second highest value being a 4. Based on the theoretical implications of this variable, we decided not to recode it as a log or square root transformation, especially since bringing this value down in range was not logical given that the second highest value was one below it.

Model Fit

To examine the fit of the Sexual Domain Model, we entered the variables associated with sexual preoccupation, sexualized coping, self-represented sexual deviancy, age, prior sexual charges, number of deviant victims, number of sexual victims, prior or index child pornography offenses, and number of male victims, to predict the latent variable of ECWC. We first freely entered these variables, allowing the model to estimate the values of each parameter. The variance of the age variable was 200 times the size of the variance for the other variables. We re-scaled this variable by multiplying it by 0.1 to ensure accurate model fit with our all parameters (Hallquist, 2018). With this model specification, we examined an RMSEA of 0.143, SRMR of 0.109, CFI of 0.683, and a TLI of 0.517. Overall, this indicates poor model fit; however, not all of the parameters were being predicted accurately according to the Sexual Domain model. Specifically, age was predicted as being positively associated with ECWC, whereas under the Sexual Domain model, age is hypothesized as being negatively associated with ECWC. Similarly, prior or index child pornography offences, though a non-significant parameter, was predicted as being negatively associated with ECWC, where under the Sexual Domain model, we would expect it to be positively associated with ECWC.

To examine the fit of the Sexual Domain Model more accurately with our data, we fixed the parameter of age to be negative, and the parameter of prior or index child pornography offences to be positive. The fit indices again indicated poor model fit (See Table 2).

The Psychological Immaturity Model

Assumptions

To assess for multivariate normality, we again used the E-statistic Energy test, examined the multivariate skewness and kurtosis values, and examined Q-Q plots for each variable. The E-

statistic and skewness and kurtosis values were all non-significant, indicating multivariate normality. We again examined Q-Q plots based on generated distributions according to each variable's sample size, mean, and standard deviation, which all followed normal distributions. We assessed for outliers using the Grubbs test, which revealed no high or low outliers on any of the variables.

Model Fit

To examine the fit of the Psychological Immaturity Model, we entered the variables associated with age (recoded), problem solving ability, tenor of intimate relationships, capacity for an intimate relationship, social influences, lack of concern, impulsivity, and education, to predict the latent variable of ECWC. We first freely entered these variables, allowing the model to estimate the values of each parameter. For consistency, we included the re-scaled version of the age variable instead of the original variable, as we did in the Sexual Domain model. With this model specification, we examined an RMSEA of 0.118, SRMR of 0.079, CFI of 0.771, and a TLI of 0.679. Overall, this indicates poor model fit; however, all of the parameters were being predicted opposite from what is specified under the Psychological Immaturity model. Specifically, age and level of education were predicted as being positively associated with ECWC, whereas under the Psychological Immaturity model, we would expect them to both be negatively associated with ECWC. Similarly, problem solving ability, tenor of intimate relationships, capacity for intimate relationships, social influences, lack of concern, and impulsivity were all predicted as being negatively associated with ECWC, whereas under the Psychological Immaturity model, we would expect all of these factors to be positively associated with ECWC.

To examine the fit of the Psychological Immaturity more accurately with our data, we fixed all of the parameters. Age and education were each fixed to be negative, and problem-solving ability, tenor of intimate relationships, capacity for intimate relationships, social influences, lack of concern, and impulsivity were each fixed to be positive. The model indices for the respecified model again indicated relatively poor fit (see Table 2).

Freely Entered Model

Due to the poor model fit for all three of our models, we decided to examine how the model would fit when all variables were freely entered into one grand model.

Assumptions

Using the same 983 participants, we found multivariate normality across the variables and a lack of outliers using the same methods as described above. In this model, we included the variables from all three models; specifically, loneliness, relationship stability, sexual deviancy, cooperation with supervision, impulsivity, hostility to women, sexual preoccupation, sexualized coping, self-represented sexual deviancy, age, prior sexual charges, number of deviant victims, number of sexual victims, child pornography charges, number of male victims, issues with problem solving, tenor of intimate relationships, capacity for intimate relationships, negative sexual influences, lack of concern, and education.

With these 21 predictors, we have 42 free parameters to estimate. The df of our model is 189, indicating that we have an over-identified model. For a participant to parameter ratio of 20:1 with 42 free parameters, we require a minimum of 840 participants. We have 983 participants included in our model estimation; therefore, we have met this assumption.

Model Fit

To examine the fit of this freely entered model, we allowed all of our predictors to be freely estimated. For consistency, we again included the re-scaled version of the age variable. With this model specification, we examined an RMSEA of 0.185, an SRMR of 0.134, a CFI of 0.261, and a TLI of 0.179. Overall, this indicates poor model fit; however, all parameters are now moving in the correct direction, as identified by each of the models. Only the child pornography charges variable was non-significant ($p = .977$). See Table 2.

Model Comparisons

To compare the model fit of these four models, we used the Vuong test (1989). Compared to the Sexual Domain and Psychological Immaturity models, the Blockage model showed significantly better fit ($p < .001$) with our sample. Compared to the Psychological Immaturity model, the Sexual Domain model showed significantly better fit ($p < .001$) with our sample. Additionally, the freely estimated model showed significantly worse fit compared to all three models of ECWC ($p < .001$).

We also generated 95% confidence intervals comparing the AIC and BIC values associated with each model. In general, a comparatively smaller AIC or BIC value indicates a better model fit. The Blockage model had significantly lower AIC and BIC values compared to both the Sexual Domain and the Psychological Immaturity models, as indicated by their 95% confidence intervals not containing 0. The Sexual Domain model additionally had significantly lower AIC and BIC values compared to the Psychological Immaturity model. All three models of ECWC had significantly lower AIC and BIC values compared to the freely estimated model. Overall, this suggests that the Blockage model provided the best fit in explaining ECWC in our sample, with the Sexual Domain model additionally providing better fit than the Psychological

Immaturity model, and all models of ECWC providing better fit than a freely entered model. That said, all models had poor fit indices suggesting that another model may better describe the data.

Model Adjustments

As demonstrated, we found relatively poor model fit for all three models of ECWC, as well as the freely entered model. With such poor model fit, we chose to re-specify the models, including only superordinate variables for the different correlates of ECWC. Specifically, we retained only the Sexual Deviancy item of the STABLE-2007, but removed the Self-Represented Sexual Deviancy, Number of Deviant Victims, and Number of Sexual Victims items, which are subordinate items of the Sexual Deviancy item. Similarly, we retained only the Lack of Relationship Stability item of the STABLE-2007, but removed the Capacity for an Intimate Relationship, and Tenor of Intimate Relationships items, which are subordinate items of the Lack of Relationship Stability item. All assumptions remained satisfied under this model specification. The results from these analyses are reported in the main paper.

PART B: LCA

We used LCA to assess for subgroups of men high in ECWC who have a history of SOC. The following correlates were included as indicator variables in the LCA: loneliness, relationship instability, sexual deviancy, child pornography charges, lack of compliance with authority figures, impulsivity, hostility toward women, sexual pre-occupation, sexualized coping, offender age, number of male victims, poor cognitive problem-solving ability, level of education, lack of concern, and negative social influences. This leads to 16 indicator variables.

Assumptions

LCA does not require assumptions of linearity, normality, or homogeneity of variance. The data must be categorical or ordinal, and the model should be either just- or over-identified. The indicator variables should have local independence – specifically, that the association between the observed variables is *explained by* the class of the latent variables. As of now, there is no specified required number of participants necessary to conduct an LCA (Weller et al., 2020); however, there is a general consensus of a "more is better" rule, with Nylund-Gibson and Choi (2018) suggesting 300 as ideal. We included 377 participants in our LCAs, excluding any participants who had a score of 0 on the ECWC item of the STABLE-2007 (*n* = 704; see participant exclusion criteria in main paper). There is, again, no determined number of required indicator variables (Weller et al., 2020), but a general consensus, again, that more leads to better results (Wurpts & Geiser, 2014). We had 16 indicator variables.

To conduct an LCA, a decision must be made of whether indicator variables will be recoded or analyzed with their original response options. Polytomous variables (i.e., a variable with more than two distinct categories) can be dichotomized or reduced to smaller sets of options, for example, examining specifically high compared to low sexual interest in children,

rather than high, moderate, and low. Recoding multiple response options into two or three options allows for better interpretation of the class solution. To meet these specifications, we recoded all STABLE-2007 variables as "high" or "low," with scores of 1 and 2 making up the "high" category, and scores of 0 making up the "low" category. The Sexual Deviancy item of the STABLE-2007 was not coded this way; we excluded any participants with a score of 0 on this item from any of our analyses, so we instead had scores of 1 on this item making up the "low" category and scores of 2 making up the "high" category. The education variable was recoded to include those who had finished high school in the "high" category, and those who had not in the "low" category. The age variable was recoded to include those whose age was above the mean (m = 41) in the "low" category, and those whose age was below the mean in the "high" category. The prior sexual charges variable was recoded to include anyone with a prior sexual charge in the "high" category, and anyone without a prior sexual charge in the "low" category.

The models that we examined were all over-identified. We examined the standardized bivariate residuals between each indicator variable as a potential indicator of local dependence. Most of the standardized bivariate residuals were non-significant ($z < |1.96|$), with the exception of those between relationship stability and prior sexual charges ($z = 2.37$), cooperation with supervision and problem-solving ability ($z = 2.74$), and impulsivity and index of prior CP charges ($z = 2.45$). While these significant bivariate residuals may indicate local dependence, the covariances between each of these pairs was approximately 0, which may indicate local independence. Covariances between each other pair of indictors were similarly approximately 0.

It has numerously been argued that local independence is an unrealistic assumption in LCA, specifically with behavioural analyses (e.g., Hagenaars, 1988; Reboussin et al., 2008). Similarly, it has been argued that the assumption of local independence can be relaxed when

allowing the indicators to be freely estimated and when indicators are loading onto latent variable(s) only (e.g., Hagenaars, 1988; Lee et al., 2020). Given that our models were not improved with the addition of another class, we have kept the three-factor solution, despite an indicator of local dependence.

Results

A three-class solution emerged as best fitting with our data. We have included the table outlining class association with each predictor in our main paper. In Table 3, we have outlined how the predictors are associated with each class, as well as colour coded to compare these classes with the three models of ECWC.

Table 3

Three-Class Solution of Subgroups of Men With ECWC

	Class		
Predictor	1	2	3
Loneliness		High	High
Relationship Instability	High	High	High
Sexual Deviancy		Low	High
Lack of Cooperation with Supervision	Low	Low	
Impulsivity	Low		High
Hostility Toward Women	Low	Low	
Sexual Preoccupation		Low	High
Sexualized Coping	Low	Low	High
Young Age	Low	High	High
Prior Sexual Charges	Low	Low	
Prior or Index CP Charges	Low	Low	Low
Many Male Victims	Low	Low	Low
Poor Cognitive Problem Solving		High	High
Negative Social Influence	Low		High
Lack of Concern for Others	Low	Low	High
Lack of High School Completion	Low	High	

Note. **Blue** = same as blockage model. **Green** = same as sexual domain model. **Purple** = same as psychological immaturity model. No colour indicates that this predictor is being endorsed opposite of what is predicted by that model.

We determine the number of classes to retain based on BIC and AIC criterion comparisons, the Lo-Mendell-Rubin likelihood ratio test (LMR-LRT), and goodness of fit indices (see Table 4). Compared to the two-class solution, the three-class solution had a slightly higher BIC value, but a substantially smaller AIC value. Goodness of fit indices were much smaller for the three-class compared to the two-class solution. Additionally, the LMR-LRT indicated that the three-class solution had statistically better fit than the two-class solution. Compared to the three-class solution, the four-class solution had a higher BIC, and a slightly smaller AIC. Goodness of fit indices were slightly smaller for the four-class compared to the three-class solution. The LMR-LRT also indicated that the four-class solution had statistically better fit than the three-class solution. We saw the same pattern of results for each additional class added to the model.

For this reason, we computed elbow plots to examine where the BIC and AIC values leveled out to determine the accurate number of classes to retain. Between the models with two and three classes, there was a very small BIC value change, with each subsequent BIC increase being substantially larger. Between the models with two and three classes, there was a large AIC value decrease, with each subsequent AIC decrease being substantially smaller. Both elbow plots indicate that the BIC and AIC values level out at the three-class solution (see Figure 1). The three-class solution additionally has more substantive interpretability and utility than the two-class solution; therefore, we have retained the three-class solution.

To check for the local independence assumption, we examined bivariate residuals between each of the included correlates for the three-class solution. Most residuals were below |1.96|, although three were slightly above. We chose to examine the standardized covariance matrix to further assess for dependency between these variables. All covariances were

approximately zero, which indicates weak relationships between our variables. We additionally examined bivariate Pearson correlations between each of the correlates. All correlations were relatively small (largest $r = 0.39$); therefore, we continued with the LCA.

Table 4

Fit Indices for LCA Class Decisions

Classes	BIC	AIC	LMR-LRT	X^2	G^2
Two	7278.58	7148.81		87293.77	2534.86
Three	7282.38	7085.76	97.05*	64197.87	2440.54
Four	7328.87	7065.41	54.36*	61440.10	2400.67

Note. BIC = Bayesian information criterion AIC = Akaike information criterion; LMR-LRT = Lo-Mendell-Rubin likelihood ratio test.

$*p < .001$.

Figure 1

Elbow Plots of AIC and BIC Values for each LCA Class Increase

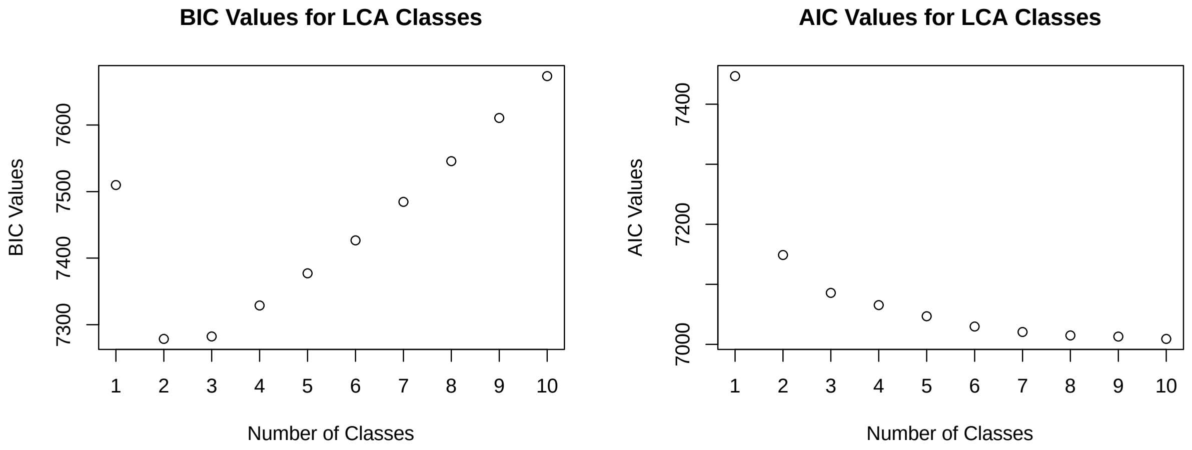

Part C: Cox Regression

Assumptions

Cox regression requires that the survival curves for different strata have hazard functions that are proportional over time. We examined Schoenfeld residuals for each analysis, all of which indicated proportional hazards (all $p > .05$). Cox regression additionally requires that the relationship between the predictor and risk scores is linear. Specifically, that for each one-unit increase in the predictor, the relative risk increases by a fixed and equal amount. We examined this using a goodness of fit test, which is similar to the Hosmer and Lemeshow test for logistic regression. The goodness of fit test assesses the fit between the observed recidivism rates and those predicted by the Cox regression. Goodness of fit tests for each analysis were non-significant, which indicates a linear relationship. Harrell's *C* is nonparametric and does not assume proportional hazards. Risk score should not be categorized as "high" and "low" risk, but rather by maintaining their actual values.

References

Hagenaars, J. A. (1988). Latent structure models with direct effects between indicators: Local dependence models. *Sociological Methods & Research, 16*(3), 379–405. https://doi.org/10.1177/0049124188016003002

Hallquist, M. (2018, August 15). *Introductory SEM using lavaan.* Penn State University, Psychology. https://psu-psychology.github.io/r-bootcamp-2018/talks/lavaan_tutorial.html.

Lee, J., Jung, K. & Park, J. (2020). Detecting conditional dependence using flexible Bayesian latent class analysis. *Frontier in Psychology,* 11:1987. https://doi.org/10.3389/fpsyg.2020.01987

Nylund-Gibson, K., & Choi, A. Y. (2018). Ten frequently asked questions abouts latent class analysis. *Translational Issues in Psychological Science, 4*(4), 440-461. https://doi.org/10.1037/tps0000176

Reboussin, B. A., Ip, E. H., & Wolfson, M. (2008). Locally dependent latent class models with covariates: an application to under-age drinking in the USA. *Journal of the Royal Statistical Society. Series A, (Statistics in Society), 171*(4), 877–897. https://doi.org/10.1111/j.1467-985X.2008.00544.x

Weller, B. E., Bowen, N. K., & Faubert, S. J. (2020). Latent Class Analysis: A Guide to Best Practice. *Journal of Black Psychology*, *46*(4), 287-311. https://doi.org/10.1177/0095798420930932

Wurpts, I. C., & Geiser, C. (2014). Is adding more indicators to a latent class analysis beneficial or detrimental? Results of a Monte Carlo study. *Frontiers in Psychology*, 5, 1-15. https://doi.org/10.3389/fpsyg.2014.00920

PART C

SPSS Syntax

```
* Encoding: UTF-8.
***Fraser, J. M., Babchishin, K. M., Helmus, L. M. (2022).
***ECWC models.

*use with dataset "COPY_ECWC dataset"

*FOR SEM.

*create a variable that identifies cases with Ndevvictims_07.1 = 0
    plus with Ndevvictims_07.1 = 0 with St99_08 = 1 AND (solicitation = 0 OR
    solicitationconviction_priors = 0 OR solicitationcharge_priors = 0).

USE ALL.

RECODE solicitation (MISSING = 0) (ELSE = 1) INTO R_solicitation.
VARIABLE LABELS R_solicitation 'R_solicitation: recode solicitation to include 0'.
VALUE LABELS R_solicitation 0 'No online solicitation' 1 'Online solicitation'.
EXECUTE.

FREQUENCIES VARIABLES=emotionalID_07.1 Ndevvictims_07.1 St99_08 R_solicitation
    solicitationconviction_priors solicitationcharge_priors
  /ORDER=ANALYSIS.

IF (Ndevvictims_07.1 = 0) dontinclude = 1.
IF ((Ndevvictims_07.1 > 1) AND (St99_08 = 1) AND ((R_solicitation = 0) AND
        (solicitationconviction_priors = 0) AND (solicitationcharge_priors = 0))) dontinclude =
        2.
VARIABLE LABELS dontinclude 'dontinclude: identify participant to not include in analysis'.
VALUE LABELS dontinclude 1 'No dev vics' 2 'Non-contact and no internet offence'.
EXECUTE.

FREQUENCIES VARIABLES= dontinclude
  /ORDER=ANALYSIS.

RECODE dontinclude (MISSING = 0) (ELSE = 1) INTO include.
VARIABLE LABELS include 'include: identify participant to include in analysis'.
VALUE LABELS include 1 'Dont include' 0 'Include'.
EXECUTE.

FREQUENCIES VARIABLES= include
  /ORDER=ANALYSIS.
```

```
*filter out cases with include = 1 AND no emotionalID_07.1 score.
USE ALL.
COMPUTE filter_$=(include = 0 & emotionalID_07.1 >= 0).
VARIABLE LABELS filter_$ 'include = 0 (FILTER)'.
VALUE LABELS filter_$ 0 'Not Selected' 1 'Selected'.
FORMATS filter_$ (f1.0).
FILTER BY filter_$.
EXECUTE.

FREQUENCIES VARIABLES=emotionalID_07.1 Ndevvictims_07.1 St99_08 R_solicitation
    solicitationconviction_priors solicitationcharge_priors
  /ORDER=ANALYSIS.


*create a variable that identifies cases with missing data on any of correlates or emotional
        ID_07.1.

*Blockage model.
RECODE loneliness.1 (MISSING = 0) (ELSE = 1) INTO m_loneliness.1.
VARIABLE LABELS m_loneliness.1 'm_loneliness.1: identify participants with missing data on
        loneliness.1'.
VALUE LABELS m_loneliness.1 0 'Missing' 1 'Not missing'.
EXECUTE.

FREQUENCIES VARIABLES= loneliness.1 m_loneliness.1
  /ORDER=ANALYSIS.

RECODE relationship_07.1 (MISSING = 0) (ELSE = 1) INTO m_relationship_07.1.
VARIABLE LABELS m_relationship_07.1 'm_relationship_07.1: identify participants with
        missing data on relationship_07.1'.
VALUE LABELS m_relationship_07.1 0 'Missing' 1 'Not missing'.
EXECUTE.

FREQUENCIES VARIABLES= relationship_07.1 m_relationship_07.1
  /ORDER=ANALYSIS.

RECODE deviantsex_07.1 (MISSING = 0) (ELSE = 1) INTO m_deviantsex_07.1.
VARIABLE LABELS m_deviantsex_07.1 'm_deviantsex_07.1: identify participants with
        missing data on deviantsex_07.1'.
VALUE LABELS m_deviantsex_07.1 0 'Missing' 1 'Not missing'.
EXECUTE.

FREQUENCIES VARIABLES= deviantsex_07.1 m_deviantsex_07.1
  /ORDER=ANALYSIS.
```

```
RECODE coopsup.1 (MISSING = 0) (ELSE = 1) INTO m_coopsup.1.
VARIABLE LABELS m_coopsup.1 'm_coopsup.1: identify participants with missing data on
        coopsup.1'.
VALUE LABELS m_coopsup.1 0 'Missing' 1 'Not missing'.
EXECUTE.

FREQUENCIES VARIABLES= coopsup.1 m_coopsup.1
  /ORDER=ANALYSIS.

RECODE impulsive.1 (MISSING = 0) (ELSE = 1) INTO m_impulsive.1.
VARIABLE LABELS m_impulsive.1 'm_impulsive.1: identify participants with missing data on
        impulsive.1'.
VALUE LABELS m_impulsive.1 0 'Missing' 1 'Not missing'.
EXECUTE.

FREQUENCIES VARIABLES= impulsive.1 m_impulsive.1
  /ORDER=ANALYSIS.

RECODE hostilewomen.1 (MISSING = 0) (ELSE = 1) INTO m_hostilewomen.1.
VARIABLE LABELS m_hostilewomen.1 'm_hostilewomen.1: identify participants with missing
        data on hostilewomen.1'.
VALUE LABELS m_hostilewomen.1 0 'Missing' 1 'Not missing'.
EXECUTE.

FREQUENCIES VARIABLES= hostilewomen.1 m_hostilewomen.1
  /ORDER=ANALYSIS.

RECODE emotionalID_07.1 (MISSING = 0) (ELSE = 1) INTO m_emotionalID_07.1.
VARIABLE LABELS m_emotionalID_07.1 'm_emotionalID_07.1: identify participants with
        missing data on emotionalID_07.1'.
VALUE LABELS m_emotionalID_07.1 0 'Missing' 1 'Not missing'.
EXECUTE.

FREQUENCIES VARIABLES= emotionalID_07.1 m_emotionalID_07.1
  /ORDER=ANALYSIS.

*create variable that groups these together.
IF (m_loneliness.1 = 0) blockmiss = 1.
IF (m_relationship_07.1 = 0) blockmiss = 1.
IF (m_deviantsex_07.1 = 0) blockmiss = 1.
IF (m_coopsup.1 = 0) blockmiss = 1.
IF (m_impulsive.1 = 0) blockmiss = 1.
IF (m_hostilewomen.1 = 0) blockmiss = 1.
IF (m_emotionalID_07.1 = 0) blockmiss = 1.
VARIABLE LABELS blockmiss 'blockmiss: identify participants with missing data for
        blockage model'.
```

```
VALUE LABELS blockmiss 1 'Missing' 0 'Not missing'.
EXECUTE.

RECODE blockmiss (MISSING = 0) (ELSE = 1).
EXECUTE.

FREQUENCIES VARIABLES= blockmiss
  /ORDER=ANALYSIS.


*check with filters.
USE ALL.
COMPUTE filter_$=(include = 0).
VARIABLE LABELS filter_$ 'include = 0 (FILTER)'.
VALUE LABELS filter_$ 0 'Not Selected' 1 'Selected'.
FORMATS filter_$ (f1.0).
FILTER BY filter_$.
EXECUTE.

FREQUENCIES VARIABLES= blockmiss
  /ORDER=ANALYSIS.

USE ALL.
COMPUTE filter_$=(include = 0 AND blockmiss = 0).
VARIABLE LABELS filter_$ 'include = 0 AND blockmiss = 0(FILTER)'.
VALUE LABELS filter_$ 0 'Not Selected' 1 'Selected'.
FORMATS filter_$ (f1.0).
FILTER BY filter_$.
EXECUTE.

FREQUENCIES VARIABLES= loneliness.1 relationship_07.1 deviantsex_07.1 coopsup.1
        impulsive.1 hostilewomen.1 emotionalID_07.1
  /ORDER=ANALYSIS.



*Sexual Domain model.
RECODE sexpreocc.1 (MISSING = 0) (ELSE = 1) INTO m_sexpreocc.1.
VARIABLE LABELS m_sexpreocc.1 'm_sexpreocc.1: identify participants with missing data on
        sexpreocc.1'.
VALUE LABELS m_sexpreocc.1 0 'Missing' 1 'Not missing'.
EXECUTE.

FREQUENCIES VARIABLES= sexpreocc.1 m_sexpreocc.1
  /ORDER=ANALYSIS.
```

```
RECODE sexcope.1 (MISSING = 0) (ELSE = 1) INTO m_sexcope.1.
VARIABLE LABELS m_sexcope.1 'm_sexcope.1: identify participants with missing data on
        sexcope.1'.
VALUE LABELS m_sexcope.1 0 'Missing' 1 'Not missing'.
EXECUTE.

FREQUENCIES VARIABLES= sexcope.1 m_sexcope.1
  /ORDER=ANALYSIS.

RECODE selfrepdev_07.1 (MISSING = 0) (ELSE = 1) INTO m_selfrepdev_07.1.
VARIABLE LABELS m_selfrepdev_07.1 'm_selfrepdev_07.1: identify participants with
        missing data on selfrepdev_07.1'.
VALUE LABELS m_selfrepdev_07.1 0 'Missing' 1 'Not missing'.
EXECUTE.

FREQUENCIES VARIABLES=  selfrepdev_07.1 m_selfrepdev_07.1
  /ORDER=ANALYSIS.

RECODE age (MISSING = 0) (ELSE = 1) INTO m_age.
VARIABLE LABELS m_age 'm_age: identify participants with missing data on age'.
VALUE LABELS m_age 0 'Missing' 1 'Not missing'.
EXECUTE.

FREQUENCIES VARIABLES= age m_age
  /ORDER=ANALYSIS.

RECODE sexcharge_priors (MISSING = 0) (ELSE = 1) INTO m_sexcharge_priors.
VARIABLE LABELS m_sexcharge_priors 'm_sexcharge_priors: identify participants with
        missing data on sexcharge_priors'.
VALUE LABELS m_sexcharge_priors 0 'Missing' 1 'Not missing'.
EXECUTE.

FREQUENCIES VARIABLES= sexcharge_priors m_sexcharge_priors
  /ORDER=ANALYSIS.

RECODE Ndevvictims_07.1 (MISSING = 0) (ELSE = 1) INTO m_Ndevvictims_07.1.
VARIABLE LABELS m_Ndevvictims_07.1 'm_sNdevvictims_07.1: identify participants with
        missing data on Ndevvictims_07.1'.
VALUE LABELS m_Ndevvictims_07.1 0 'Missing' 1 'Not missing'.
EXECUTE.

FREQUENCIES VARIABLES= Ndevvictims_07.1 m_Ndevvictims_07.1
  /ORDER=ANALYSIS.

RECODE Nsexvictims_07.1 (MISSING = 0) (ELSE = 1) INTO m_Nsexvictims_07.1.
```

```
VARIABLE LABELS m_Nsexvictims_07.1 'm_Nsexvictims_07.1: identify participants with
        missing data on Nsexvictims_07.1'.
VALUE LABELS m_Nsexvictims_07.1 0 'Missing' 1 'Not missing'.
EXECUTE.

FREQUENCIES VARIABLES= Nsexvictims_07.1 m_Nsexvictims_07.1
  /ORDER=ANALYSIS.

RECODE CP_PriorORindex (MISSING = 0) (ELSE = 1) INTO m_CP_PriorORindex.
VARIABLE LABELS m_CP_PriorORindex 'm_CP_PriorORindex: identify participants with
        missing data on CP_PriorORindex'.
VALUE LABELS m_CP_PriorORindex 0 'Missing' 1 'Not missing'.
EXECUTE.

FREQUENCIES VARIABLES= CP_PriorORindex m_CP_PriorORindex
  /ORDER=ANALYSIS.

RECODE St99_11 (MISSING = 0) (ELSE = 1) INTO m_St99_11.
VARIABLE LABELS m_St99_11 'm_St99_11: identify participants with missing data on
        St99_11'.
VALUE LABELS m_St99_11 0 'Missing' 1 'Not missing'.
EXECUTE.

FREQUENCIES VARIABLES= St99_11 m_St99_11
  /ORDER=ANALYSIS.

IF ((m_sexpreocc.1 = 0) OR (m_sexcope.1 = 0) OR (m_selfrepdev_07.1 = 0) OR (m_age = 0)
    OR (m_sexcharge_priors = 0) OR (m_Ndevvictims_07.1 = 0) OR (m_Nsexvictims_07.1 = 0)
    OR (m_CP_PriorORindex = 0) OR (m_St99_11 = 0) OR (m_emotionalID_07.1 = 0))
        sexualmiss = 1.
VARIABLE LABELS sexualmiss 'sexualmiss: identify participants with missing data for sexual
        domain model'.
VALUE LABELS sexualmiss 1 'Missing' 0 'Not missing'.
EXECUTE.

RECODE sexualmiss (MISSING = 0) (ELSE = 1).
EXECUTE.

FREQUENCIES VARIABLES= sexualmiss
  /ORDER=ANALYSIS.

USE ALL.
COMPUTE filter_$=(include = 0 AND sexualmiss = 0).
VARIABLE LABELS filter_$ 'include = 0 AND sexual = 0(FILTER)'.
VALUE LABELS filter_$ 0 'Not Selected' 1 'Selected'.
FORMATS filter_$ (f1.0).
```

```
FILTER BY filter_$.
EXECUTE.

FREQUENCIES VARIABLES= emotionalID_07.1
  /ORDER=ANALYSIS.


*Psychological Immaturity model.
USE ALL.

RECODE probsolve.1 (MISSING = 0) (ELSE = 1) INTO m_probsolve.1.
VARIABLE LABELS m_probsolve.1 'm_probsolve.1: identify participants with missing data on
        probsolve.1'.
VALUE LABELS m_probsolve.1 0 'Missing' 1 'Not missing'.
EXECUTE.

FREQUENCIES VARIABLES= probsolve.1 m_probsolve.1
  /ORDER=ANALYSIS.

RECODE tenorrelp_07.1 (MISSING = 0) (ELSE = 1) INTO m_tenorrelp_07.1.
VARIABLE LABELS m_tenorrelp_07.1 'm_tenorrelp_07.1: identify participants with missing
        data on tenorrelp_07.1'.
VALUE LABELS m_tenorrelp_07.1 0 'Missing' 1 'Not missing'.
EXECUTE.

FREQUENCIES VARIABLES= tenorrelp_07.1 m_tenorrelp_07.1
  /ORDER=ANALYSIS.

RECODE rel2years_07.1 (MISSING = 0) (ELSE = 1) INTO m_rel2years_07.1.
VARIABLE LABELS m_rel2years_07.1 'm_rel2years_07.1: identify participants with missing
        data on rel2years_071'.
VALUE LABELS m_rel2years_07.1 0 'Missing' 1 'Not missing'.
EXECUTE.

FREQUENCIES VARIABLES= rel2years_07.1 m_rel2years_07.1
  /ORDER=ANALYSIS.

RECODE socinfl.1 (MISSING = 0) (ELSE = 1) INTO m_socinfl.1.
VARIABLE LABELS m_socinfl.1 'm_socinfl.1: identify participants with missing data on
        socinfl.1'.
VALUE LABELS m_socinfl.1 0 'Missing' 1 'Not missing'.
EXECUTE.

FREQUENCIES VARIABLES= socinfl.1 m_tsocinfl.1
  /ORDER=ANALYSIS.
```

```
RECODE lackconcern.1 (MISSING = 0) (ELSE = 1) INTO m_lackconcern.1.
VARIABLE LABELS m_lackconcern.1 'm_lackconcern.1: identify participants with missing
        data on lackconcern.1'.
VALUE LABELS m_lackconcern.1 0 'Missing' 1 'Not missing'.
EXECUTE.

FREQUENCIES VARIABLES= lackconcern.1 m_lackconcern.1
  /ORDER=ANALYSIS.

RECODE Education (MISSING = 0) (ELSE = 1) INTO m_Education.
VARIABLE LABELS m_Education 'm_Education: identify participants with missing data on
        Education1'.
VALUE LABELS m_Education 0 'Missing' 1 'Not missing'.
EXECUTE.

FREQUENCIES VARIABLES= Education m_Education
  /ORDER=ANALYSIS.

IF ((m_probsolve.1 = 0) OR (m_tenorrelp_07.1 = 0) OR (m_rel2years_07.1 = 0) OR
        (m_socinfl.1 = 0) OR (m_age = 0)
    OR (m_lackconcern.1 = 0) OR (m_Education = 0) OR (m_impulsive.1= 0)
    OR (m_emotionalID_07.1 = 0)) psychmiss = 1.
VARIABLE LABELS psychmiss 'psychmiss: identify participants with missing data for
        psychological immaturity model'.
VALUE LABELS psychmiss 1 'Missing' 0 'Not missing'.
EXECUTE.

RECODE psychmiss (MISSING = 0) (ELSE = 1).
EXECUTE.

FREQUENCIES VARIABLES= psychmiss
  /ORDER=ANALYSIS.

USE ALL.
COMPUTE filter_$=(include = 0 AND psychmiss = 0).
VARIABLE LABELS filter_$ 'include = 0 AND psychmiss = 0(FILTER)'.
VALUE LABELS filter_$ 0 'Not Selected' 1 'Selected'.
FORMATS filter_$ (f1.0).
FILTER BY filter_$.
EXECUTE.

FREQUENCIES VARIABLES= emotionalID_07.1
  /ORDER=ANALYSIS.
```

```
*create grand missing data variable to account for those with missing data across the three
        models.

IF ((blockmiss = 0) AND (sexualmiss = 0) AND (psychmiss = 0)) missingtot = 1.
EXECUTE.

RECODE missingtot (1=1) (ELSE = 0).
VARIABLE LABELS missingtot 'identify participants with missing data on any sem model'.
VALUE LABELS missingtot 1 'Not missing' 0 'Missing'.
EXECUTE.

USE ALL.
COMPUTE filter_$=(include = 0 AND missingtot = 1).
VARIABLE LABELS filter_$ 'include = 0 AND missingtot = 1(FILTER)'.
VALUE LABELS filter_$ 0 'Not Selected' 1 'Selected'.
FORMATS filter_$ (f1.0).
FILTER BY filter_$.
EXECUTE.

FREQUENCIES VARIABLES= emotionalID_07.1
  /ORDER=ANALYSIS.



*get demographic information.
USE ALL.
COMPUTE filter_$=(include = 0 AND missingtot = 1).
VARIABLE LABELS filter_$ 'include = 0 AND missingtot = 1(FILTER)'.
VALUE LABELS filter_$ 0 'Not Selected' 1 'Selected'.
FORMATS filter_$ (f1.0).
FILTER BY filter_$.
EXECUTE.

DESCRIPTIVES VARIABLES= age Race Education Stat99Rriskcate STABLE07_proxy.1
        sexcharge_priors
  /STATISTICS=MEAN STDDEV RANGE MIN MAX KURTOSIS SKEWNESS.

FREQUENCIES VARIABLES = age Race Education Stat99Rriskcate STABLE07_proxy.1
        sexcharge_priors
  /STATISTICS=MEAN MEDIAN MODE
  /ORDER=ANALYSIS.



*create combined STABLE 2007 and static 99r variable.
```

```
IF (Stat99Rriskcate <= 1) AND (STABLE07_proxy.1 <= 3) combinedrisk = 1.
IF (Stat99Rriskcate <= 1) AND (4 <= STABLE07_proxy.1 <= 11) combinedrisk = 1.
IF (Stat99Rriskcate <= 1) AND (STABLE07_proxy.1 >= 12) combinedrisk = 2.
IF ((Stat99Rriskcate = 2) OR (Stat99Rriskcate = 3)) AND (STABLE07_proxy.1 <= 3)
        combinedrisk = 1.
IF ((Stat99Rriskcate = 2) OR (Stat99Rriskcate = 3)) AND (4 <= STABLE07_proxy.1 <= 11)
        combinedrisk = 2.
IF ((Stat99Rriskcate = 2) OR (Stat99Rriskcate = 3)) AND (STABLE07_proxy.1 >= 12)
        combinedrisk = 3.
IF ((Stat99Rriskcate = 4) OR (Stat99Rriskcate = 5)) AND (STABLE07_proxy.1 <= 3)
        combinedrisk = 2.
IF ((Stat99Rriskcate = 4) OR (Stat99Rriskcate = 5)) AND (4 <= STABLE07_proxy.1 <= 11)
        combinedrisk = 3.
IF ((Stat99Rriskcate = 4) OR (Stat99Rriskcate = 5)) AND (STABLE07_proxy.1 >= 12)
        combinedrisk = 4.
IF (Stat99Rriskcate >= 6) AND (STABLE07_proxy.1 <= 3) combinedrisk = 4.
IF (Stat99Rriskcate >= 6) AND (4 <= STABLE07_proxy.1 <= 11) combinedrisk = 4.
IF (Stat99Rriskcate >= 6) AND (STABLE07_proxy.1 >= 12) combinedrisk = 5.
VARIABLE LABELS combinedrisk 'combinedrisk: Combined STABLE-2007 and Static-99R
        risk'.
VALUE LABELS combinedrisk
   1 'low'
   2 'moderate-low'
   3 'moderate-high'
   4 'high'
   5 'very high'.
EXECUTE.

USE ALL.
COMPUTE filter_$=(include = 0 AND missingtot = 1).
VARIABLE LABELS filter_$ 'include = 0 AND missingtot = 1(FILTER)'.
VALUE LABELS filter_$ 0 'Not Selected' 1 'Selected'.
FORMATS filter_$ (f1.0).
FILTER BY filter_$.
EXECUTE.

DESCRIPTIVES VARIABLES=combinedrisk
  /STATISTICS=MEAN STDDEV MIN MAX KURTOSIS SKEWNESS.

FREQUENCIES VARIABLES=combinedrisk
  /STATISTICS=MEAN MEDIAN MODE
  /ORDER=ANALYSIS.
```

```
*FOR LCA.

*recode vars to be dichotomous.
if (age < 41) dichot_age = 1.
if (age >= 41) dichot_age = 0.
variable labels dichot_age 'Dichotomize age variable'.
value labels dichot_age 1 'Young' 0 'Not young'.
execute.

if (sexcharge_priors = 0) dichot_sexcharge_priors = 0.
if (sexcharge_priors >= 1) dichot_sexcharge_priors = 1.
variable labels dichot_sexcharge_priors 'Dichotomize sexcharg_priors'.
value labels dichot_sexcharge_priors 0 'No priors' 1 'At least one prior'.
execute.

if (Education = 1) dichot_Education = 0.
if (Education = 2) dichot_Education = 0.
if (Education = 3) dichot_Education = 0.
if (Education = 4) dichot_Education = 0.
if (Education = 5) dichot_Education = 1.
if (Education = 6) dichot_Education = 1.
if (Education = 7) dichot_Education = 1.
variable labels dichot_Education 'Dichotomize Education'.
value labels dichot_Education 0 'Did not finish hs' 1 'Finished hs'.
execute.

*recode all variables to be dichotomous with 1s and 2s only.
RECODE loneliness.1 (0 = 1) (1 = 2) (2 = 2) into r_loneliness.1.
VARIABLE LABELS r_loneliness.1 'recode loneliness.1 for LCA'.
EXECUTE.

FREQUENCIES VARIABLES=loneliness.1 r_loneliness.1
  /STATISTICS=MEAN MEDIAN MODE
  /ORDER=ANALYSIS.

RECODE relationship_07.1 (0 = 1) (1 = 2) (2 = 2) into r_relationship_07.1.
VARIABLE LABELS r_relationship_07.1 'recode relationship_07.1 for LCA'.
EXECUTE.

FREQUENCIES VARIABLES=relationship_07.1 r_relationship_07.1
  /STATISTICS=MEAN MEDIAN MODE
  /ORDER=ANALYSIS.

RECODE deviantsex_07.1 (0 = 1) (1 = 2) (2 = 2) into r_deviantsex_07.1.
VARIABLE LABELS r_deviantsex_07.1 'recode deviantsex_07.1 for LCA'.
EXECUTE.
```

```
FREQUENCIES VARIABLES=deviantsex_07.1 r_deviantsex_07.1
  /STATISTICS=MEAN MEDIAN MODE
  /ORDER=ANALYSIS.

RECODE coopsup.1 (0 = 1) (1 = 2) (2 = 2) into r_coopsup.1.
VARIABLE LABELS r_coopsup.1 'recode coopsup.1 for LCA'.
EXECUTE.

FREQUENCIES VARIABLES=coopsup.1 r_coopsup.1
  /STATISTICS=MEAN MEDIAN MODE
  /ORDER=ANALYSIS.

RECODE impulsive.1 (0 = 1) (1 = 2) (2 = 2) into r_impulsive.1.
VARIABLE LABELS r_impulsive.1 'recode impulsive.1 for LCA'.
EXECUTE.

FREQUENCIES VARIABLES=impulsive.1 r_impulsive.1
  /STATISTICS=MEAN MEDIAN MODE
  /ORDER=ANALYSIS.

RECODE hostilewomen.1 (0 = 1) (1 = 2) (2 = 2) into r_hostilewomen.1.
VARIABLE LABELS r_hostilewomen.1 'recode hostilewomen.1 for LCA'.
EXECUTE.

FREQUENCIES VARIABLES=hostilewomen.1 r_hostilewomen.1
  /STATISTICS=MEAN MEDIAN MODE
  /ORDER=ANALYSIS.

RECODE sexpreocc.1 (0 = 1) (1 = 2) (2 = 2) into r_sexpreocc.1.
VARIABLE LABELS r_sexpreocc.1 'recode sexpreocc.1 for LCA'.
EXECUTE.

FREQUENCIES VARIABLES=sexpreocc.1 r_sexpreocc.1
  /STATISTICS=MEAN MEDIAN MODE
  /ORDER=ANALYSIS.

RECODE sexcope.1 (0 = 1) (1 = 2) (2 = 2) into r_sexcope.1.
VARIABLE LABELS r_sexcope.1 'recode sexcope.1 for LCA'.
EXECUTE.

FREQUENCIES VARIABLES=sexcope.1 r_sexcope.1
  /STATISTICS=MEAN MEDIAN MODE
  /ORDER=ANALYSIS.

RECODE selfrepdev_07.1 (0 = 1) (1 = 2) (2 = 2) into r_selfrepdev_07.1.
```

```
VARIABLE LABELS r_selfrepdev_07.1 'recode selfrepdev_07.1 for LCA'.
EXECUTE.

FREQUENCIES VARIABLES=selfrepdev_07.1 r_selfrepdev_07.1
  /STATISTICS=MEAN MEDIAN MODE
  /ORDER=ANALYSIS.

RECODE dichot_age (0 = 1) (1 = 2) into r_dichot_age.
VARIABLE LABELS r_dichot_age 'recode dichot_age for LCA'.
EXECUTE.

FREQUENCIES VARIABLES=dichot_age r_dichot_age
  /STATISTICS=MEAN MEDIAN MODE
  /ORDER=ANALYSIS.

RECODE dichot_sexcharge_priors (0 = 1) (1 = 2) into r_dichot_sexcharge_priors.
VARIABLE LABELS r_dichot_sexcharge_priors 'recode dichot_sexcharge_priosr for LCA'.
EXECUTE.

FREQUENCIES VARIABLES=dichot_sexcharge_priors r_dichot_sexcharge_priors
  /STATISTICS=MEAN MEDIAN MODE
  /ORDER=ANALYSIS.

RECODE Ndevvictims_07.1 (0 = 1) (1 = 2) (2 = 2) into r_Ndevvictims_07.1.
VARIABLE LABELS r_Ndevvictims_07.1 'recode Ndevvictims_07.1 for LCA'.
EXECUTE.

FREQUENCIES VARIABLES=Ndevvictims_07.1 r_Ndevvictims_07.1
  /STATISTICS=MEAN MEDIAN MODE
  /ORDER=ANALYSIS.

RECODE Nsexvictims_07.1 (0 = 1) (1 = 2) (2 = 2) into r_Nsexvictims_07.1.
VARIABLE LABELS r_Nsexvictims_07.1 'recode Nsexvictims_07.1 for LCA'.
EXECUTE.

FREQUENCIES VARIABLES=Nsexvictims_07.1 r_Nsexvictims_07.1
  /STATISTICS=MEAN MEDIAN MODE
  /ORDER=ANALYSIS.

RECODE CP_PriorORindex (0 = 1) (1 = 2) into r_CP_PriorORindex.
VARIABLE LABELS r_CP_PriorORindex 'recode CP_PriorORindex for LCA'.
EXECUTE.

FREQUENCIES VARIABLES=CP_PriorORindex r_CP_PriorORindex
  /STATISTICS=MEAN MEDIAN MODE
  /ORDER=ANALYSIS.
```

```
RECODE St99_11 (0 = 1) (1 = 2) into r_St99_11.
VARIABLE LABELS r_St99_11 'recode St99_11 for LCA'.
EXECUTE.

FREQUENCIES VARIABLES=St99_11 r_St99_11
  /STATISTICS=MEAN MEDIAN MODE
  /ORDER=ANALYSIS.

RECODE probsolve.1 (0 = 1) (1 = 2) (2 = 2) into r_probsolve.1.
VARIABLE LABELS r_probsolve.1 'recode probsolve.1 for LCA'.
EXECUTE.

FREQUENCIES VARIABLES=probsolve.1 r_probsolve.1
  /STATISTICS=MEAN MEDIAN MODE
  /ORDER=ANALYSIS.

RECODE tenorrelp_07.1 (0 = 1) (1 = 2) (2 = 2) into r_tenorrelp_07.1.
VARIABLE LABELS r_tenorrelp_07.1 'recode tenorrelp_07.1 for LCA'.
EXECUTE.

FREQUENCIES VARIABLES=tenorrelp_07.1 r_tenorrelp_07.1
  /STATISTICS=MEAN MEDIAN MODE
  /ORDER=ANALYSIS.

RECODE rel2years_07.1 (0 = 1) (1 = 2) into r_rel2years_07.1.
VARIABLE LABELS r_rel2years_07.1 'recode rel2years_07.1 for LCA'.
EXECUTE.

FREQUENCIES VARIABLES=rel2years_07.1 r_rel2years_07.1
  /STATISTICS=MEAN MEDIAN MODE
  /ORDER=ANALYSIS.

RECODE socinfl.1 (0 = 1) (1 = 2) (2 = 2) into r_socinfl.1.
VARIABLE LABELS r_socinfl.1 'recode socinfl.1 for LCA'.
EXECUTE.

FREQUENCIES VARIABLES=socinfl.1 r_socinfl.1
  /STATISTICS=MEAN MEDIAN MODE
  /ORDER=ANALYSIS.

RECODE lackconcern.1 (0 = 1) (1 = 2) (2 = 2) into r_lackconcern.1.
VARIABLE LABELS r_lackconcern.1 'recode lackconcern.1 for LCA'.
EXECUTE.

FREQUENCIES VARIABLES=lackconcern.1 r_lackconcern.1
```

```
  /STATISTICS=MEAN MEDIAN MODE
  /ORDER=ANALYSIS.

RECODE dichot_Education (0 = 1) (1 = 2) into r_dichot_Education.
VARIABLE LABELS r_dichot_Education 'recode dichot_Education for LCA'.
EXECUTE.

FREQUENCIES VARIABLES=dichot_Education r_dichot_Education
  /STATISTICS=MEAN MEDIAN MODE
  /ORDER=ANALYSIS.




*AUC.
USE ALL.
COMPUTE filter_$=(include = 0 AND missingtot = 1).
VARIABLE LABELS filter_$ 'include = 0 AND missingtot = 1(FILTER)'.
VALUE LABELS filter_$ 0 'Not Selected' 1 'Selected'.
FORMATS filter_$ (f1.0).
FILTER BY filter_$.
EXECUTE.

ROC emotionalID_07.1 BY St99_11 (1)
  /PLOT=CURVE
  /PRINT=SE
  /CRITERIA=CUTOFF(INCLUDE) TESTPOS(LARGE) DISTRIBUTION(FREE) CI(95)
  /MISSING=EXCLUDE.

CORRELATIONS
  /VARIABLES=St99_11 emotionalID_07.1
  /PRINT=TWOTAIL NOSIG FULL
  /STATISTICS DESCRIPTIVES
  /MISSING=PAIRWISE.
```

R Script

```
#Fraser, J. M., Babchishin, K. M., Helmus, L. M. (2022)
#ECWC models

#import dataset.
setwd("Desktop")
data <- read.csv(file = "COPY_ECWC dataset_2022-03-17.csv")


#load packages for SEM.
library(lavaan)
library(dplyr)
library(psych)
library(QuantPsyc)
library(energy)
library(outliers)
library(performance)
library(nonnest2)
library(tidySEM)
library(ggplot2)
library(qgraph)
library(semPlot)


#filter out any missing data (see SPSS syntax)
ecwc <- filter(data, include == 0, missingtot == 1)

####BLOCKAGE MODEL####

#check for normality and outliers.

#create variables to save descriptive stats of each correlate in Blockage model.
loneliness_descrip <- describe(ecwc$loneliness.1)
relationship_descrip <- describe(ecwc$relationship_07.1)
deviantsex_descrip <- describe(ecwc$deviantsex_07.1)
coopsup_descrip <- describe(ecwc$coopsup.1)
impulsive_descrip <- describe(ecwc$impulsive.1)
hostilewomen_descrip <- describe(ecwc$hostilewomen.1)

#create a data frame of descriptives.
normblock <- data.frame(x1 = rnorm(loneliness_descrip$n, loneliness_descrip$mean,
        loneliness_descrip$sd),
                x2 = rnorm(relationship_descrip$n, relationship_descrip$mean,
        relationship_descrip$sd),
```

```
        x3 = rnorm(deviantsex_descrip$n, deviantsex_descrip$mean,
    deviantsex_descrip$sd),
        x4 = rnorm(coopsup_descrip$n, coopsup_descrip$mean, coopsup_descrip$sd),
        x5 = rnorm(impulsive_descrip$n, impulsive_descrip$mean,
    impulsive_descrip$sd),
        x6 = rnorm(hostilewomen_descrip$n, hostilewomen_descrip$mean,
    hostilewomen_descrip$sd))
#create a data frame for all correlates of Blockage model.
normblock2 <- data.frame(x1 = rnorm(ecwc$loneliness.1),
                         x2 = rnorm(ecwc$relationship_07.1),
                         x3 = rnorm(ecwc$deviantsex_07.1),
                         x4 = rnorm(ecwc$coopsup.1),
                         x5 = rnorm(ecwc$impulsive.1),
                         x6 = rnorm(ecwc$hostilewomen.1))

#test for normality with descriptives data frame.
set.seed(0)
mult.norm(normblock)$mult.test
set.seed(0)
mvnorm.etest(normblock , 100)

#test for normality with model data frame.
set.seed(0)
mult.norm(normblock2)$mult.test
set.seed(0)
mvnorm.etest(normblock2, 100)

#examine qq plots of descriptive variables.
qqnorm(rnorm(loneliness_descrip$n, loneliness_descrip$mean, loneliness_descrip$sd))
qqnorm(rnorm(relationship_descrip$n, relationship_descrip$mean, relationship_descrip$sd))
qqnorm(rnorm(deviantsex_descrip$n, deviantsex_descrip$mean, deviantsex_descrip$sd))
qqnorm(rnorm(coopsup_descrip$n, coopsup_descrip$mean, coopsup_descrip$sd))
qqnorm(rnorm(impulsive_descrip$n, impulsive_descrip$mean, impulsive_descrip$sd))
qqnorm(rnorm(hostilewomen_descrip$n, hostilewomen_descrip$mean,
    hostilewomen_descrip$sd))

#test for outliers.
grubbs.test(ecwc$loneliness.1, opposite = TRUE)
grubbs.test(ecwc$loneliness.1)
grubbs.test(ecwc$relationship_07.1, opposite = TRUE)
grubbs.test(ecwc$relationship_07.1)
grubbs.test(ecwc$deviantsex_07.1, opposite = TRUE)
grubbs.test(ecwc$deviantsex_07.1)
grubbs.test(ecwc$coopsup.1, opposite = TRUE)
grubbs.test(ecwc$coopsup.1)
grubbs.test(ecwc$impulsive.1, opposite = TRUE)
```

```
grubbs.test(ecwc$impulsive.1)
grubbs.test(ecwc$hostilewomen.1, opposite = TRUE)
grubbs.test(ecwc$hostilewomen.1)

#look at covariance matrix.
block <- data.frame(
  loneliness = ecwc$loneliness.1,
  relationship = ecwc$relationship_07.1,
  deviantsex = ecwc$deviantsex_07.1,
  coopsup = ecwc$coopsup.1,
  impuslive = ecwc$impulsive.1,
  hostilewomen = ecwc$hostilewomen.1)
cov(block)

#sem equation.
blockeq <- 'emoID =~ loneliness.1 + relationship_07.1 + deviantsex_07.1 + coopsup.1 +
        impulsive.1 + hostilewomen.1'
blockfit <- sem(model = blockeq , data = ecwc, std.lv = TRUE)
summary(blockfit, fit.measures = TRUE, rsquare = TRUE)

#number of parameters.
coef(blockfit)
lavInspect(blockfit, 'list')

#fix variables to correct direction.
blockeq2 <- 'emoID =~ a*loneliness.1 + b*relationship_07.1 + c*deviantsex_07.1 +
        d*coopsup.1 + e*impulsive.1 + f*hostilewomen.1
a > 0.15
b > 0.15
c < -0.15
d > 0.15
e < -0.2
f > 0.15'

blockfit2 <- sem(model = blockeq2 , data = ecwc, std.lv = TRUE)
blockfit2summ <- summary(blockfit2, fit.measures = TRUE, rsquare = TRUE)



####SEXUAL DOMAIN MODEL####

#check for normality and outliers.

#create descriptive variables for correlates of Sexual Domain model.
sexpreocc_descrip <- describe(ecwc$sexpreocc.1)
sexcope_descrip <- describe(ecwc$sexcope.1)
```

```
age_descrip <- describe(ecwc$age)
sexcharge_priors_descrip <- describe(ecwc$sexcharge_priors)
CP_descrip <- describe(ecwc$CP_PriorORindex)
malevics_descrip <- describe(ecwc$St99_11)
#deviant sex descriptive already made from Blockage model.

#create data frame of descriptives.
normsex <- data.frame(x1 = rnorm(sexpreocc_descrip$n, sexpreocc_descrip$mean,
        sexpreocc_descrip$sd),
                x2 = rnorm(sexcope_descrip$n, sexcope_descrip$mean, sexcope_descrip$sd),
                x3 = rnorm(age_descrip$n, age_descrip$mean, age_descrip$sd),
                x4 = rnorm(deviantsex_descrip$n, deviantsex_descrip$mean,
        deviantsex_descrip$sd),
                x5 = rnorm(sexcharge_priors_descrip$n, sexcharge_priors_descrip$mean,
        sexcharge_priors_descrip$sd),
                x6 = rnorm(CP_descrip$n, CP_descrip$mean, CP_descrip$sd),
                x7 = rnorm(malevics_descrip$n, malevics_descrip$mean, malevics_descrip$sd))
#create data frame of correlates.
normsex2 <- data.frame(x1 = rnorm(ecwc$sexpreocc.1),
                 x2 = rnorm(ecwc$sexcope.1),
                 x3 = rnorm(ecwc$age),
                 x4 = rnorm(ecwc$deviantsex_07.1),
                 x5 = rnorm(ecwc$sexcharge_priors),
                 x6 = rnorm(ecwc$CP_PriorORindex),
                 x7 = rnorm(ecwc$St99_11))

#check for normality with descriptives data frame.
set.seed(0)
mult.norm(normsex)$mult.test
set.seed(0)
mvnorm.etest(normsex , 100)

#check for normality with correlates data frame.
set.seed(0)
mult.norm(normsex2)$mult.test
set.seed(0)
mvnorm.etest(normsex2, 100)

#look at qq plots with descriptives.
qqnorm(rnorm(sexpreocc_descrip$n, sexpreocc_descrip$mean, sexpreocc_descrip$sd))
qqnorm(rnorm(sexcope_descrip$n, sexcope_descrip$mean, sexcope_descrip$sd))
qqnorm(rnorm(age_descrip$n, age_descrip$mean, age_descrip$sd))
qqnorm(rnorm(sexcharge_priors_descrip$n, sexcharge_priors_descrip$mean,
        sexcharge_priors_descrip$sd))
qqnorm(rnorm(CP_descrip$n, CP_descrip$mean, CP_descrip$sd))
qqnorm(rnorm(malevics_descrip$n, malevics_descrip$mean, malevics_descrip$sd))
```

```
#check for outliers.
grubbs.test(ecwc$sexpreocc.1, opposite = TRUE)
grubbs.test(ecwc$sexpreocc.1)
grubbs.test(ecwc$sexcope.1, opposite = TRUE)
grubbs.test(ecwc$sexcope.1)
grubbs.test(ecwc$age, opposite = TRUE)
grubbs.test(ecwc$age)
grubbs.test(ecwc$sexcharge_priors, opposite = TRUE)
grubbs.test(ecwc$sexcharge_priors)
grubbs.test(ecwc$CP_PriorORindex, opposite = TRUE)
grubbs.test(ecwc$CP_PriorORindex)
grubbs.test(ecwc$St99_11, opposite = TRUE)
grubbs.test(ecwc$St99_11)

#covariance matrix.
sex <- data.frame(
  sexpreocc = ecwc$sexpreocc.1,
  sexcope = ecwc$sexcope.1,
  deviantsex = ecwc$deviantsex_07.1,
  age = ecwc$age,
  sexchargepriors = ecwc$sexcharge_priors,
  CP = ecwc$CP_PriorORindex,
  Nmalevictims = ecwc$St99_11)
cov(sex)

#SEM equation.
sexeq <- 'emoID =~ sexpreocc.1 + sexcope.1 + deviantsex_07.1 + age + sexcharge_priors +
          CP_PriorORindex + St99_11'

sexfit <- sem(sexeq, data=ecwc, std.lv = TRUE)
summary(sexfit, fit.measures = TRUE, rsquare = TRUE)
#age variance is about 200 times the size of the others.
#multiply it by 0.1 to rescale it.

ecwc$rage <- ecwc$age * 0.1

#new equation.
sexeq <- 'emoID =~ sexpreocc.1 + sexcope.1 + deviantsex_07.1 + rage + sexcharge_priors +
          CP_PriorORindex + St99_11'

sexfit <- sem(sexeq, data=ecwc, std.lv = TRUE)
summary(sexfit, fit.measures = TRUE, rsquare = TRUE)

#number of free parameters.
lavInspect(sexfit, 'list')
```

```
####PSYCHOLOGICAL IMMATURITY MODEL####

#check for normality and outliers.

#save descriptive variables for the correlates of the Psych Immaturity model.
probsolve_descrip <- describe(ecwc$probsolve.1)
socinfl_descrip <- describe(ecwc$socinfl.1)
lackconern_descrip <- describe(ecwc$lackconcern.1)
education_descrip <- describe(ecwc$Education)
#age descrip already exists
#relationship descrip already exists.
#impulsive already exists.

#create data frame of descriptive variables.
normpsych <- data.frame(x1 = rnorm(age_descrip$n, age_descrip$mean, age_descrip$sd),
        x2 = rnorm(probsolve_descrip$n, probsolve_descrip$mean,
    probsolve_descrip$sd),
        x4 = rnorm(socinfl_descrip$n, socinfl_descrip$mean, socinfl_descrip$sd),
        x5 = rnorm(lackconern_descrip$n, lackconern_descrip$mean,
    lackconern_descrip$sd),
        x6 = rnorm(impulsive_descrip$n, impulsive_descrip$mean,
    impulsive_descrip$sd),
        x7 = rnorm(education_descrip$n, education_descrip$mean,
    education_descrip$sd),
        x8 = rnorm(relationship_descrip$n, relationship_descrip$mean,
    relationship_descrip$sd))
#create data frame of correlates.
normpsych2 <- data.frame(x1 = rnorm(ecwc$rage),
        x2 = rnorm(ecwc$probsolve.1),
        x4 = rnorm(ecwc$socinfl.1),
        x5 = rnorm(ecwc$lackconcern.1),
        x6 = rnorm(ecwc$impulsive.1),
        x7 = rnorm(ecwc$Education),
        x8 = rnorm(ecwc$relationship_07.1))

#test for normality with descriptives.
set.seed(0)
mult.norm(normpsych)$mult.test
set.seed(0)
mvnorm.etest(normpsych , 100)

#test for normality with correlates.
set.seed(0)
```

```
mult.norm(normpsych2)$mult.test
set.seed(0)
mvnorm.etest(normpsych2 , 100)

#look at qq plots with descriptives.
qqnorm(rnorm(age_descrip$n, age_descrip$mean, age_descrip$sd))
qqnorm(rnorm(probsolve_descrip$n, probsolve_descrip$mean, probsolve_descrip$sd))
qqnorm(rnorm(socinfl_descrip$n, socinfl_descrip$mean, socinfl_descrip$sd))
qqnorm(rnorm(lackconern_descrip$n, lackconern_descrip$mean, lackconern_descrip$sd))
qqnorm(rnorm(impulsive_descrip$n, impulsive_descrip$mean, impulsive_descrip$sd))
qqnorm(rnorm(education_descrip$n, education_descrip$mean, education_descrip$sd))

#test for outliers.
grubbs.test(ecwc$age, opposite = TRUE)
grubbs.test(ecwc$age)
grubbs.test(ecwc$probsolve.1, opposite = TRUE)
grubbs.test(ecwc$probsolve.1)
grubbs.test(ecwc$socinfl.1, opposite = TRUE)
grubbs.test(ecwc$socinfl.1)
grubbs.test(ecwc$lackconcern.1, opposite = TRUE)
grubbs.test(ecwc$lackconcern.1)
grubbs.test(ecwc$impulsive.1, opposite = TRUE)
grubbs.test(ecwc$impulsive.1)
grubbs.test(ecwc$Education, opposite = TRUE)
grubbs.test(ecwc$Education)

#covariance matrix.
psych <- data.frame(
  age = ecwc$rage,
  probsolve = ecwc$probsolve.1,
  relationship = ecwc$relationship_07.1,
  socinfl = ecwc$socinfl.1,
  lackconcern = ecwc$lackconcern.1,
  impulsive = ecwc$impulsive.1,
  education = ecwc$Education)
cov(psych)

#SEM equation.
psycheq <- 'emoID =~ rage + probsolve.1 + relationship_07.1 + socinfl.1 + lackconcern.1 +
        impulsive.1 + Education'

psychfit <- sem(psycheq, data=ecwc, std.lv = TRUE)
summary(psychfit, fit.measures = TRUE, rsquare = TRUE)

#number of free parameters.
lavInspect(psychfit, 'list')
```

```
#fix everything to correct direction.
psycheq2 <- 'emoID =~ a*rage + b*probsolve.1 + c*relationship_07.1 + d*socinfl.1 +
        e*lackconcern.1 + f*impulsive.1 + g*Education
a < 0
b > 0
c > 0
d > 0
e > 0
f > 0
g < 0'

psychfit2 <- sem(psycheq2, data=ecwc, std.lv = TRUE)
psychfit2summ <- summary(psychfit2, fit.measures = TRUE, rsquare = TRUE)


#####ENTER ALL VARS MODEL#####

#create data frame of all correlates.
fullecwc_norm1 <- data.frame(
  loneliness = rnorm(ecwc$loneliness.1),
  relationship = rnorm(ecwc$relationship_07.1),
  deviantsex = rnorm(ecwc$deviantsex_07.1),
  coopsup = rnorm(ecwc$coopsup.1),
  impuslive = rnorm(ecwc$impulsive.1),
  hostilewomen = rnorm(ecwc$hostilewomen.1),
  sexpreocc = rnorm(ecwc$sexpreocc.1),
  sexcope = rnorm(ecwc$sexcope.1),
  age = rnorm(ecwc$rage),
  sexchargepriors = rnorm(ecwc$sexcharge_priors),
  CP = rnorm(ecwc$CP_PriorORindex),
  Nmalevictims = rnorm(ecwc$St99_11),
  probsolve = rnorm(ecwc$probsolve.1),
  socinfl = rnorm(ecwc$socinfl.1),
  lackconcern = rnorm(ecwc$lackconcern.1),
  education = rnorm(ecwc$Education))

#test for normality.
set.seed(0)
mult.norm(fullecwc_norm1)$mult.test
set.seed(0)
mvnorm.etest(fullecwc_norm1 , 100)

#SEM equation.
```

```
fullecwc_eq1 <- 'emoID =~ loneliness.1 + relationship_07.1 + deviantsex_07.1 + coopsup.1 +
        impulsive.1 + hostilewomen.1 + sexpreocc.1 + sexcope.1 + rage + sexcharge_priors +
        CP_PriorORindex + St99_11 + probsolve.1 + socinfl.1 + lackconcern.1 + Education'

fullecwcfit1 <- sem(fullecwc_eq1, data=ecwc, std.lv = TRUE)
summary(fullecwcfit1, fit.measures = TRUE, rsquare = TRUE)

#number of free parameters.
lavInspect(fullecwcfit1, 'list')



#####JUST SEXUAL DEVIANCY MODEL#####

#test for normality.
set.seed(0)
mult.norm(rnorm(ecwc$deviantsex_07.1))$mult.test
set.seed(0)
mvnorm.etest(rnorm(ecwc$deviantsex_07.1) , 100)

#SEM equation.
sexdeveq <- 'emoID =~ deviantsex_07.1
deviantsex_07.1 ~~ 0*deviantsex_07.1'

devsexfit <- sem(sexdeveq, data=ecwc, std.lv = TRUE)
summary(devsexfit, fit.measures = TRUE, rsquare = TRUE)



#####MODEL COMPARISONS#####
library(nonnest2)

#compare all models using Vuong test.
vuongtest(blockfit2, sexfit)
vuongtest(blockfit2, psychfit2)
vuongtest(sexfit, psychfit2)
vuongtest(blockfit2, fullecwcfit1)
vuongtest(sexfit, fullecwcfit1)
vuongtest(psychfit2, fullecwcfit1)

vuongtest(devsexfit,blockfit2)
vuongtest(devsexfit,sexfit)
vuongtest(devsexfit,psychfit2)
vuongtest(devsexfit,fullecwcfit1)
```

```
#get CIs for the difference in AIC and BIC statistics between each model.
icci(blockfit2, sexfit)
icci(blockfit2, psychfit2)
icci(sexfit, psychfit2)
icci(blockfit2, fullecwcfit1)
icci(sexfit, fullecwcfit1)
icci(psychfit2, fullecwcfit1)

icci(devsexfit, blockfit2)
icci(devsexfit, sexfit)
icci(devsexfit, psychfit2)
icci(devsexfit, fullecwcfit1)




#####LATENT CLASS ANALYSIS#####
library(poLCA)
library(tidyLPA)
library(Hmisc)

#import data set (see SPSS syntax for coding of new dataset)
data2 <- read.csv(file = "COPY_ECWC dataset_2022-03-17.csv")

#include only participants who meet exclusion criteria and who have ECWC score greater than
    0.
ecwc2 <- filter(data2, include == 0, emotionalID_07.1 > 0)

#LCA formula.
f1 <- as.formula(cbind(r_loneliness.1, r_relationship_07.1, deviantsex_07.1, r_coopsup.1,
    r_impulsive.1,
        r_hostilewomen.1, r_sexpreocc.1, r_sexcope.1,
        r_dichot_age, r_dichot_sexcharge_priors, r_CP_PriorORindex, r_St99_11,
    r_probsolve.1,
        r_socinfl.1, r_lackconcern.1,r_dichot_Education)~1)
#create data frame of all LCA variables.
f3 <- data.frame(ecwc2$r_loneliness.1,
        ecwc2$r_relationship_07.1,
        ecwc2$deviantsex_07.1,
        ecwc2$r_coopsup.1,
        ecwc2$r_impulsive.1,
        ecwc2$r_hostilewomen.1,
        ecwc2$r_sexpreocc.1,
        ecwc2$r_sexcope.1,
        ecwc2$r_dichot_age,
        ecwc2$r_dichot_sexcharge_priors,
```

```
        ecwc2$r_CP_PriorORindex,
        ecwc2$r_St99_11,
        ecwc2$r_probsolve.1,
        ecwc2$r_socinfl.1,
        ecwc2$r_lackconcern.1,
        ecwc2$r_dichot_Education)

#run LCAs, starting with 1 class, then 2, then 3, etc.
set.seed(0)
lca <- poLCA(f1, data = ecwc2, nclass = 1, nrep = 100, na.rm = FALSE)

set.seed(0)
lca1 <- poLCA(f1, data = ecwc2, nclass = 2, nrep = 100, na.rm = FALSE)
poLCA.entropy(lca1)
#compare 1 and 2 class solution with LMR-LRT.
lcav1 <- calc_lrt(lca$Nobs, lca$llik, lca$npar, 1, lca1$llik, lca1$npar, 2)

set.seed(0)
lca2 <- poLCA(f1, data = ecwc2, nclass = 3, nrep = 100, na.rm = FALSE)
poLCA.entropy(lca2)
#compare 2 and 3 class solution.
lca1v2 <- calc_lrt(lca1$Nobs, lca1$llik, lca1$npar, 2, lca2$llik, lca2$npar, 3)

set.seed(0)
lca3 <- poLCA(f1, data = ecwc2, nclass = 4, nrep = 100, na.rm = FALSE)
poLCA.entropy(lca3)
#compare 3 and 4 class solution.
lca2v3 <- calc_lrt(lca2$Nobs, lca2$llik, lca2$npar, 3, lca3$llik, lca3$npar, 4)

set.seed(0)
lca4 <- poLCA(f1, data = ecwc2, nclass = 5, nrep = 100, na.rm = FALSE)
#compare 4 and 5 class solution.
lca3v4 <- calc_lrt(lca3$Nobs, lca3$llik, lca3$npar, 4, lca4$llik, lca4$npar, 5)

set.seed(0)
lca5 <- poLCA(f1, data = ecwc2, nclass = 6, nrep = 100, na.rm = FALSE)
#compare 5 and 6 class solution.
lca4v5 <- calc_lrt(lca4$Nobs, lca4$llik, lca4$npar, 5, lca5$llik, lca5$npar, 6)

set.seed(0)
lca6 <- poLCA(f1, data = ecwc2, nclass = 7, nrep = 100, na.rm = FALSE)
#compare 6 and 7 class solution.
lca5v6 <- calc_lrt(lca5$Nobs, lca5$llik, lca5$npar, 6, lca6$llik, lca6$npar, 7)

set.seed(0)
lca7 <- poLCA(f1, data = ecwc2, nclass = 8, nrep = 100, na.rm = FALSE)
```

```
#compare 7 and 8 class solution.
lca6v7 <- calc_lrt(lca6$Nobs, lca6$llik, lca6$npar, 7, lca7$llik, lca7$npar, 8)

set.seed(0)
lca8 <- poLCA(f1, data = ecwc2, nclass = 9, nrep = 100, na.rm = FALSE)
#compare 8 and 9 class solution.
lca7v8 <- calc_lrt(lca7$Nobs, lca7$llik, lca7$npar, 8, lca8$llik, lca8$npar, 9)

set.seed(0)
lca9 <- poLCA(f1, data = ecwc2, nclass = 10, nrep = 100, na.rm = FALSE)
#compare 9 and 10 class solution.
lca8v9 <- calc_lrt(lca8$Nobs, lca8$llik, lca8$npar, 9, lca9$llik, lca9$npar, 10)


#look at plots of AICs and BICs for 1-10 class solutions.
aicvals <- data.frame(lca$aic,
                  lca1$aic,
                  lca2$aic,
                  lca3$aic,
                  lca4$aic,
                  lca5$aic,
                  lca6$aic,
                  lca7$aic,
                  lca8$aic,
                  lca9$aic)
plot(1:10, aicvals, main = "AIC Values for LCA Classes",
    xlab = "Number of Classes", ylab = "AIC Values", xaxt = "n")
axis(1, at = seq(1, 10, by = 1), las = 1)

bicvals <- data.frame(lca$bic,
                  lca1$bic,
                  lca2$bic,
                  lca3$bic,
                  lca4$bic,
                  lca5$bic,
                  lca6$bic,
                  lca7$bic,
                  lca8$bic,
                  lca9$bic)
plot(1:10, bicvals, main = "BIC Values for LCA Classes",
    xlab = "Number of Classes", ylab = "BIC Values", xaxt = "n")
axis(1, at = seq(1, 10, by = 1), las = 1)



#check for local independence in three class solution.
```

```
#create a variable to identify class association in the dataset.
lca2_predclass <- lca2$predclass
ecwc2$classID <- lca2_predclass

#get and examine bivariate residuals.
bvr <- function(lca2) {
  stopifnot(class(lca2) == 'poLCA')

  ov_names <- names(lca2$predcell)[1:(ncol(lca2$predcell) - 2)]
  ov_combn <- combn(ov_names, 2)

  get_bvr <- function(ov_pair) {
    form_obs <- as.formula(paste0('observed ~', ov_pair[1], "+", ov_pair[2]))
    form_exp <- as.formula(paste0("expected ~", ov_pair[1], "+", ov_pair[2]))

    counts_obs <- xtabs(form_obs, data = lca2$predcell)
    counts_exp <- xtabs(form_exp, data = lca2$predcell)

    bvr <- sum((counts_obs - counts_exp)^2 / counts_exp)

    bvr
  }

  bvr_pairs <- apply(ov_combn, 2, get_bvr)
  attr(bvr_pairs, "class") <- "dist"
  attr(bvr_pairs, "Size") <- length(ov_names)
  attr(bvr_pairs, "Labels") <- ov_names
  attr(bvr_pairs, "Diag") <- FALSE
  attr(bvr_pairs, "Upper") <- FALSE

  bvr_pairs
}

bi_resids <- bvr(lca2)
bi_resids_stand <- scale(bi_resids)

#look at covariance matrix.
covlca <- cov(f3, use = "complete.obs")
#look at correlation matrix.
lcacors <- rcorr(as.matrix(f3), type = 'pearson')



#####COX REGRESSION#####
library(survival)
```

```
library(survminer)
library(survMisc)
library(dynpred)

#Cox Reg to see if ECWC differentially associated with recidivism in three classes.

#class 1.
class1 <- filter(ecwc2, include == 0, classID == 1)

#Any sex recidivism.
coxsex <- coxph(Surv(TimeSex, rcd_sex)~emotionalID_07.1,
                data = class1)
summary(coxsex)

#check for linear relationship between predictor and risk scores.
gof_sex <- gof(coxsex, 3)
#check for proportional hazards.
resid_sex <- cox.zph(coxsex)

HC_sex <- cindex(Surv(TimeSex, rcd_sex)~emotionalID_07.1,
              data = class1)
#calculate 95% CI.
uppersexCI <- coxsex$concordance[6] + 1.96*(coxsex$concordance[7])
lowersexCI <- coxsex$concordance[6] - 1.96*(coxsex$concordance[7])
#calculate 84% CI.
uppersexCI2 <- coxsex$concordance[6] + 1.44*(coxsex$concordance[7])
lowersexCI2 <- coxsex$concordance[6] - 1.44*(coxsex$concordance[7])


#Any recidivism.
coxAny <- coxph(Surv(TimeAny, rcd_any)~emotionalID_07.1,
             data = class1)
summary(coxAny)

#check linearity.
gof_Any <- gof(coxAny, 3)
#check proportional hazards.
resid_Any <- cox.zph(coxAny)

HC_Any <- cindex(Surv(TimeAny, rcd_any)~emotionalID_07.1,
              data = class1)
#calculate 95% CI.
upperanyCI <- coxAny$concordance[6] + 1.96*(coxAny$concordance[7])
loweranyCI <- coxAny$concordance[6] - 1.96*(coxAny$concordance[7])
#calculate 84% CI.
upperanyCI2 <- coxAny$concordance[6] + 1.44*(coxAny$concordance[7])
```

```
loweranyCI2 <- coxAny$concordance[6] - 1.44*(coxAny$concordance[7])


#Violent recidivism.
coxvio <- coxph(Surv(TimeVio, rcd_vio)~emotionalID_07.1,
             data = class1)
summary(coxvio)

#linearity.
gof_vio <- gof(coxvio, 3)
#proprtional hazards.
resid_vio <- cox.zph(coxvio)

HC_vio <- cindex(Surv(TimeVio, rcd_vio)~emotionalID_07.1,
              data = class1)
#calculate 95% CI.
uppervioCI <- coxvio$concordance[6] + 1.96*(coxvio$concordance[7])
lowervioCI <- coxvio$concordance[6] - 1.96*(coxvio$concordance[7])
#calculate 84% CI.
uppervioCI2 <- coxvio$concordance[6] + 1.44*(coxvio$concordance[7])
lowervioCI2 <- coxvio$concordance[6] - 1.44*(coxvio$concordance[7])


#Sex contact recidivism.
coxsexc <- coxph(Surv(TimeSexC, rcd_sexc)~emotionalID_07.1,
             data = class1)
summary(coxsexc)

#linearity.
gof_sexc <- gof(coxsexc, 3)
#proportional hazards.
resid_sexc <- cox.zph(coxsexc)

HC_sexc <- cindex(Surv(TimeSexC, rcd_sexc)~emotionalID_07.1,
              data = class1)
#calculate 95% CI.
uppersexcCI <- coxsexc$concordance[6] + 1.96*(coxsexc$concordance[7])
lowersexcCI <- coxsexc$concordance[6] - 1.96*(coxsexc$concordance[7])
#calculate 84% CI.
uppersexcCI2 <- coxsexc$concordance[6] + 1.44*(coxsexc$concordance[7])
lowersexcCI2 <- coxsexc$concordance[6] - 1.44*(coxsexc$concordance[7])



#CLASS 2
class2 <- filter(ecwc2, include == 0, classID == 2)
```

```
#Any sexual recidivism.
coxsex2 <- coxph(Surv(TimeSex, rcd_sex)~emotionalID_07.1,
        data = class2)
summary(coxsex2)

#linearity.
gof_sex2 <- gof(coxsex2, 3)
#proprtional hazards.
resid_sex2 <- cox.zph(coxsex2)

HC_sex2 <- cindex(Surv(TimeSex, rcd_sex)~emotionalID_07.1,
        data = class2)
#95% CI.
uppersex2CI <- coxsex2$concordance[6] + 1.96*(coxsex2$concordance[7])
lowersex2CI <- coxsex2$concordance[6] - 1.96*(coxsex2$concordance[7])
#84% CI.
uppersex2CI2 <- coxsex2$concordance[6] + 1.44*(coxsex2$concordance[7])
lowersex2CI2 <- coxsex2$concordance[6] - 1.44*(coxsex2$concordance[7])


#Any recidivism.
coxAny2 <- coxph(Surv(TimeAny, rcd_any)~emotionalID_07.1,
        data = class2)
summary(coxAny2)

#linearity.
gof_Any2 <- gof(coxAny2, 3)
#proportional hazards.
resid_Any2 <- cox.zph(coxAny2)

HC_Any2 <- cindex(Surv(TimeAny, rcd_any)~emotionalID_07.1,
        data = class2)
#95% CI.
upperany2CI <- coxAny2$concordance[6] + 1.96*(coxAny2$concordance[7])
lowerany2CI <- coxAny2$concordance[6] - 1.96*(coxAny2$concordance[7])
#84% CI.
upperany2CI2 <- coxAny2$concordance[6] + 1.44*(coxAny2$concordance[7])
lowerany2CI2 <- coxAny2$concordance[6] - 1.44*(coxAny2$concordance[7])


#Violent recidivism.
coxvio2 <- coxph(Surv(TimeVio, rcd_vio)~emotionalID_07.1,
        data = class2)
summary(coxvio2)
```

```
#linearity.
gof_vio2 <- gof(coxvio2, 3)
#proportional hazards.
resid_vio2 <- cox.zph(coxvio2)

HC_vio2 <- cindex(Surv(TimeVio, rcd_vio)~emotionalID_07.1,
               data = class2)
#95% CI.
uppervio2CI <- coxvio2$concordance[6] + 1.96*(coxvio2$concordance[7])
lowervio2CI <- coxvio2$concordance[6] - 1.96*(coxvio2$concordance[7])
#84% CI.
uppervio2CI2 <- coxvio2$concordance[6] + 1.44*(coxvio2$concordance[7])
lowervio2CI2 <- coxvio2$concordance[6] - 1.44*(coxvio2$concordance[7])


#Sexual contact recidivism.
coxsexc2 <- coxph(Surv(TimeSexC, rcd_sexc)~emotionalID_07.1,
               data = class2)
summary(coxsexc2)

#linearity.
gof_sexc2 <- gof(coxsexc2, 3)
#proprtional hazards.
resid_sexc2 <- cox.zph(coxsexc2)

HC_sexc2 <- cindex(Surv(TimeSexC, rcd_sexc)~emotionalID_07.1,
                data = class2)
#95% CI.
uppersexc2CI <- coxsexc2$concordance[6] + 1.96*(coxsexc2$concordance[7])
lowersexc2CI <- coxsexc2$concordance[6] - 1.96*(coxsexc2$concordance[7])
#84% CI.
uppersexc2CI2 <- coxsexc2$concordance[6] + 1.44*(coxsexc2$concordance[7])
lowersexc2CI2 <- coxsexc2$concordance[6] - 1.44*(coxsexc2$concordance[7])



#CLASS 3.
class3 <- filter(ecwc2, include == 0, classID == 3)

#Any sexual recidivism.
coxsex3 <- coxph(Surv(TimeSex, rcd_sex)~emotionalID_07.1,
              data = class3)
summary(coxsex3)

#linearity.
gof_sex3 <- gof(coxsex3, 3)
```

```
#proportional hazards.
resid_sex3 <- cox.zph(coxsex3)

HC_sex3 <- cindex(Surv(TimeSex, rcd_sex)~emotionalID_07.1,
              data = class3)
#95% CI.
uppersex3CI <- coxsex3$concordance[6] + 1.96*(coxsex3$concordance[7])
lowersex3CI <- coxsex3$concordance[6] - 1.96*(coxsex3$concordance[7])
#84% CI.
uppersex3CI2 <- coxsex3$concordance[6] + 1.44*(coxsex3$concordance[7])
lowersex3CI2 <- coxsex3$concordance[6] - 1.44*(coxsex3$concordance[7])


#Any recidivism.
coxAny3 <- coxph(Surv(TimeAny, rcd_any)~emotionalID_07.1,
             data = class3)
  summary(coxAny3)

#linearity.
gof_Any3 <- gof(coxAny3, 3)
#proportional hazards.
resid_Any3 <- cox.zph(coxAny3)

HC_Any3 <- cindex(Surv(TimeAny, rcd_any)~emotionalID_07.1,
              data = class3)
#95% CI.
upperany3CI <- coxAny3$concordance[6] + 1.96*(coxAny3$concordance[7])
lowerany3CI <- coxAny3$concordance[6] - 1.96*(coxAny3$concordance[7])
#84% CI.
upperany3CI2 <- coxAny3$concordance[6] + 1.44*(coxAny3$concordance[7])
lowerany3CI2 <- coxAny3$concordance[6] - 1.44*(coxAny3$concordance[7])


#Violent recidivism.
coxvio3 <- coxph(Surv(TimeVio, rcd_vio)~emotionalID_07.1,
             data = class3)
summary(coxvio3)

#linearity.
gof_vio3 <- gof(coxvio3, 3)
#proportional hazards.
resid_vio3 <- cox.zph(coxvio3)

HC_vio3 <- cindex(Surv(TimeVio, rcd_vio)~emotionalID_07.1,
              data = class3)
#95% CI.
```

```
uppervio3CI <- coxvio3$concordance[6] + 1.96*(coxvio3$concordance[7])
lowervio3CI <- coxvio3$concordance[6] - 1.96*(coxvio3$concordance[7])
#84% CI.
uppervio3CI2 <- coxvio3$concordance[6] + 1.44*(coxvio3$concordance[7])
lowervio3CI2 <- coxvio3$concordance[6] - 1.44*(coxvio3$concordance[7])


#Sexual contact recidivism.
coxsexc3 <- coxph(Surv(TimeSexC, rcd_sexc)~emotionalID_07.1,
                data = class3)
summary(coxsexc3)

#linearity.
gof_sexc3 <- gof(coxsexc3, 3)
#proportional hazards.
resid_sexc3 <- cox.zph(coxsexc3)

HC_sexc3 <- cindex(Surv(TimeSexC, rcd_sexc)~emotionalID_07.1,
                 data = class3)
#95% CI.
uppersexc3CI <- coxsexc3$concordance[6] + 1.96*(coxsexc3$concordance[7])
lowersexc3CI <- coxsexc3$concordance[6] - 1.96*(coxsexc3$concordance[7])
#84% CI.
uppersexc3CI2 <- coxsexc3$concordance[6] + 1.44*(coxsexc3$concordance[7])
lowersexc3CI2 <- coxsexc3$concordance[6] - 1.44*(coxsexc3$concordance[7])



#Cox reg to see if classes are differentially associated with recidivism.

#Any sexual recidivism.
coxreg_Sex1 <- coxph(Surv(TimeSex, rcd_sex)~relevel(factor(classID), ref = 1),
               data = ecwc2)
summary(coxreg_Sex1)
coxreg_Sex2 <- coxph(Surv(TimeSex, rcd_sex)~relevel(factor(classID), ref = 2),
                    data = ecwc2)
summary(coxreg_Sex2)
coxreg_Sex3 <- coxph(Surv(TimeSex, rcd_sex)~relevel(factor(classID), ref = 3),
                    data = ecwc2)
summary(coxreg_Sex3)

#linearity.
gof_Sex <- gof(coxreg_Sex1, 3)
#proportional hazards.
resid_Sex <- cox.zph(coxreg_Sex1)
```

```
HC_Sex1 <- cindex(Surv(TimeSex, rcd_sex)~relevel(factor(classID), ref = 3),
              data = ecwc2)
#95% CI.
highSexCI <- coxreg_Sex1$concordance[6] + 1.96*(coxreg_Sex1$concordance[7])
lowSexCI <- coxreg_Sex1$concordance[6] - 1.96*(coxreg_Sex1$concordance[7])


#Any recidivism.
coxreg_Any1 <- coxph(Surv(TimeAny, rcd_any)~relevel(factor(classID), ref = 1),
                data = ecwc2)
summary(coxreg_Any1)
coxreg_Any2 <- coxph(Surv(TimeAny, rcd_any)~relevel(factor(classID), ref = 2),
                data = ecwc2)
summary(coxreg_Any2)
coxreg_Any3 <- coxph(Surv(TimeAny, rcd_any)~relevel(factor(classID), ref = 3),
                 data = ecwc2)
summary(coxreg_Any3)

#linearity.
gof_Any <- gof(coxreg_Any1, 3)
#proportional hazards.
resid_Any <- cox.zph(coxreg_Any1)

HC_Any1 <- cindex(Surv(TimeAny, rcd_any)~relevel(factor(classID), ref = 2),
              data = ecwc2)
#95% CI.
highAnyCI <- coxreg_Any1$concordance[6] + 1.96*(coxreg_Any1$concordance[7])
lowAnyCI <- coxreg_Any1$concordance[6] - 1.96*(coxreg_Any1$concordance[7])


#Violent recidivism.
coxreg_Vio1 <- coxph(Surv(TimeVio, rcd_vio)~relevel(factor(classID), ref = 1),
                data = ecwc2)
summary(coxreg_Vio1)
coxreg_Vio2 <- coxph(Surv(TimeVio, rcd_vio)~relevel(factor(classID), ref = 2),
                data = ecwc2)
summary(coxreg_Vio2)
coxreg_Vio3 <- coxph(Surv(TimeVio, rcd_vio)~relevel(factor(classID), ref = 3),
                 data = ecwc2)
summary(coxreg_Vio3)

#linearity.
gof_Vio <- gof(coxreg_Vio, 3)
resid_Vio <- cox.zph(coxreg_Vio)

HC_Vio <- cindex(Surv(TimeVio, rcd_vio)~relevel(factor(classID), ref = 2),
```

```
                data = ecwc2)
#95% CI.
highVioCI <- coxreg_Vio1$concordance[6] + 1.96*(coxreg_Vio1$concordance[7])
lowVioCI <- coxreg_Vio1$concordance[6] - 1.96*(coxreg_Vio1$concordance[7])


#Sexual contact recidivism.
coxreg_SexC1 <- coxph(Surv(TimeSexC, rcd_sexc)~relevel(factor(classID), ref = 1),
                    data = ecwc2)
summary(coxreg_SexC1)
coxreg_SexC2 <- coxph(Surv(TimeSexC, rcd_sexc)~relevel(factor(classID), ref = 2),
                    data = ecwc2)
summary(coxreg_SexC2)
coxreg_SexC3 <- coxph(Surv(TimeSexC, rcd_sexc)~relevel(factor(classID), ref = 3),
                     data = ecwc2)
summary(coxreg_SexC3)

#linearity.
gof_SexC <- gof(coxreg_SexC, 3)
#proportional hazards.
resid_SexC <- cox.zph(coxreg_SexC)

HC_SexC <- cindex(Surv(TimeSexC, rcd_sexc)~relevel(factor(classID), ref = 2),
                 data = ecwc2)
#95% CI.
highSexCCI <- coxreg_SexC1$concordance[6] + 1.96*(coxreg_SexC1$concordance[7])
lowSexCCI <- coxreg_SexC1$concordance[6] - 1.96*(coxreg_SexC1$concordance[7])




#####RECIDIVISM DESCRIPTIVES#####

#class 1.

#create data frames for recidivism and follow-up times.
recid1 <- data.frame(x1 = class1$rcd_sex,
                    x3 = class1$rcd_any,
                    x5 = class1$rcd_vio,
                    x6 = class1$rcd_sexc)
FUrecid1 <- data.frame(x1 = class1$TimeSex,
                      x3 = class1$TimeAny,
                      x5 = class1$TimeVio,
                      x6 = class1$TimeSexC)

#descriptives.
```

```
recid1descrip <- describe(recid1)
FUrecid1descrip <- describe(FUrecid1)
mean(FUrecid1descrip$mean)

#recidivism frequencies.
recid1_sex <- table(class1$rcd_sex)
recid1_any <- table(class1$rcd_any)
recid1_vio <- table(class1$rcd_vio)
recid1_sexc <- table(class1$rcd_sexc)


#class 2

#create data frames for recidivism and follow up.
recid2 <- data.frame(x1 = class2$rcd_sex,
                     x2 = class2$rcd_any,
                     x3 = class2$rcd_vio,
                     x4 = class2$rcd_sexc)
FUrecid2 <- data.frame(x1 = class2$TimeSex,
                       x3 = class2$TimeAny,
                       x5 = class2$TimeVio,
                       x6 = class2$TimeSexC)

#descriptives.
recid2descrip <- describe(recid2)
FUrecid2descrip <- describe(FUrecid2)
mean(FUrecid2descrip$mean)

#recidivism frequencies.
recid2_sex <- table(class2$rcd_sex)
recid2_any <- table(class2$rcd_any)
recid2_vio <- table(class2$rcd_vio)
recid2_sexc <- table(class2$rcd_sexc)



#class 3
recid3 <- data.frame(x1 = class3$rcd_sex,
                     x2 = class3$rcd_any,
                     x3 = class3$rcd_vio,
                     x4 = class3$rcd_sexc)
FUrecid3 <- data.frame(x1 = class3$TimeSex,
                       x3 = class3$TimeAny,
                       x5 = class3$TimeVio,
                       x6 = class3$TimeSexC)
```

```
#descriptives.
recid3descrip <- describe(recid3)
FUrecid3descrip <- describe(FUrecid3)
mean(FUrecid3descrip$mean)

#frequencies.
recid3_sex <- table(class3$rcd_sex)
recid3_any <- table(class3$rcd_any)
recid3_vio <- table(class3$rcd_vio)
recid3_sexc <- table(class3$rcd_sexc)



#follow-up times span > 6 months.
#contain to follow up time of 2 years.

#Any sexual recidivism.
#create var to identify recidivists.
ecwc2$fixedrecidSex <- ifelse(ecwc2$TimeSex >= 2 | ecwc2$rcd_sex > 0, 1, 0)

#descriptives and frequencies.
#class1
Sex1 <- filter(ecwc2, include == 0, classID == 1, fixedrecidSex == 1)
sex1descrip <- describe(Sex1$TimeSex)
recid1_sex_fixed <- table(Sex1$rcd_sex)

#class 2.
Sex2 <- filter(ecwc2, include == 0, classID == 2, fixedrecidSex == 1)
sex2descrip <- describe(Sex2$TimeSex)
recid2_sex_fixed <- table(Sex2$rcd_sex)

#class 3.
Sex3 <- filter(ecwc2, include == 0, classID == 3, fixedrecidSex == 1)
sex3descrip <- describe(Sex3$TimeSex)
recid3_sex_fixed <- table(Sex3$rcd_sex)


#Any recidivism.
#create var to identify recidivists.
ecwc2$fixedrecidAny <- ifelse(ecwc2$TimeAny >= 2 | ecwc2$rcd_any > 0, 1, 0)

#get descriptives and frequencies.
#class 1.
Any1 <- filter(ecwc2, include == 0, classID == 1, fixedrecidAny == 1)
any1descrip <- describe(Any1$TimeAny)
recid1_any_fixed <- table(Any1$rcd_any)
```

```
#class 2.
Any2 <- filter(ecwc2, include == 0, classID == 2, fixedrecidAny == 1)
any2descrip <- describe(Any2$TimeAny)
recid2_any_fixed <- table(Any2$rcd_any)

#class 3.
Any3 <- filter(ecwc2, include == 0, classID == 3, fixedrecidAny == 1)
any3descrip <- describe(Any3$TimeAny)
recid3_any_fixed <- table(Any3$rcd_any)


#Violent recidivism.
#create var to identify recidivists.
ecwc2$fixedrecidVio <- ifelse(ecwc2$TimeVio >= 2 | ecwc2$rcd_vio > 0, 1, 0)

#descriptives and frequencies.
#class 1.
Vio1 <- filter(ecwc2, include == 0, classID == 1, fixedrecidVio == 1)
vio1descrip <- describe(Vio1$TimeVio)
recid1_Vio_fixed <- table(Vio1$rcd_vio)

#class 2.
Vio2 <- filter(ecwc2, include == 0, classID == 2, fixedrecidVio == 1)
vio2descrip <- describe(Vio2$TimeVio)
recid2_Vio_fixed <- table(Vio2$rcd_vio)

#class 3.
Vio3 <- filter(ecwc2, include == 0, classID == 3, fixedrecidVio == 1)
vio3descrip <- describe(Vio3$TimeVio)
recid3_Vio_fixed <- table(Vio3$rcd_vio)


#Sexual contact recidivism.
#create var to identify recidivists.
ecwc2$fixedrecidSexC <- ifelse(ecwc2$TimeSexC >= 2 | ecwc2$rcd_sexc > 0, 1, 0)

#descriptives and frequencies.
#class 1.
SexC1 <- filter(ecwc2, include == 0, classID == 1, fixedrecidSexC == 1)
sexc1descrip <- describe(SexC1$TimeSexC)
recid1_SexC_fixed <- table(SexC1$rcd_sexc)

SexC2 <- filter(ecwc2, include == 0, classID == 2, fixedrecidSexC == 1)
sexc2descrip <- describe(SexC2$TimeSexC)
recid2_SexC_fixed <- table(SexC2$rcd_sexc)
```

```
SexC3 <- filter(ecwc2, include == 0, classID == 3, fixedrecidSexC == 1)
sexc3descrip <- describe(SexC3$TimeSexC)
recid3_SexC_fixed <- table(SexC3$rcd_sexc)


#create new data frames with fixed follow-up times.
#separate by class.
fixedrecid1<- data.frame(x1 = sex1descrip$mean,
                         x2 = any1descrip$mean,
                         x3 = vio1descrip$mean,
                         x4 = sexc1descrip$mean)
descrip_fixedrecid1 <- describe(fixedrecid1)
mean(descrip_fixedrecid1$mean)

fixedrecid2<- data.frame(x1 = sex2descrip$mean,
                         x2 = any2descrip$mean,
                         x3 = vio2descrip$mean,
                         x4 = sexc2descrip$mean)
descrip_fixedrecid2 <- describe(fixedrecid2)
mean(descrip_fixedrecid2$mean)

fixedrecid3<- data.frame(x1 = sex3descrip$mean,
                         x2 = any3descrip$mean,
                         x3 = vio3descrip$mean,
                         x4 = sexc3descrip$mean)
descrip_fixedrecid3 <- describe(fixedrecid3)
mean(descrip_fixedrecid3$mean)


#create data sets with recidivists at fixed follow-up.

#class 1.
allfixed1 <- filter(ecwc2, include == 0, classID == 1, fixedrecidSex == 1,
                    fixedrecidAny == 1, fixedrecidSexC == 1,
                    fixedrecidVio == 1)

recid1_allfixed <- data.frame(x1 = allfixed1$rcd_sex,
                     x2 = allfixed1$rcd_any,
                     x3 = allfixed1$rcd_vio,
                     x4 = allfixed1$rcd_sexc)
describe(recid1_allfixed)

FUrecid1_fixed <- data.frame(x1 = allfixed1$TimeSex,
                       x2 = allfixed1$TimeAny,
                       x3 = allfixed1$TimeVio,
```

```
                    x4 = allfixed1$TimeSexC)
FUrecid1descrip_fixed <- describe(FUrecid1_fixed)

#descriptives.
mean(FUrecid1descrip_fixed$mean)
mean(FUrecid1descrip_fixed$sd)

#frequencies.
table(allfixed1$rcd_sex)
table(allfixed1$rcd_any)
table(allfixed1$rcd_vio)
table(allfixed1$rcd_sexc)


#class 2.
allfixed2 <- filter(ecwc2, include == 0, classID == 2, fixedrecidSex == 1,
                 fixedrecidAny == 1, fixedrecidSexC == 1,
                 fixedrecidVio == 1)

recid2_allfixed <- data.frame(x1 = allfixed2$rcd_sex,
                         x2 = allfixed2$rcd_any,
                         x3 = allfixed2$rcd_vio,
                         x4 = allfixed2$rcd_sexc)
describe(recid2_allfixed)

FUrecid2_fixed <- data.frame(x1 = allfixed2$TimeSex,
                        x2 = allfixed2$TimeAny,
                        x3 = allfixed2$TimeVio,
                        x4 = allfixed2$TimeSexC)
FUrecid2descrip_fixed <- describe(FUrecid2_fixed)

#descriptives.
mean(FUrecid2descrip_fixed$mean)
mean(FUrecid2descrip_fixed$sd)

#frequencies.
table(allfixed2$rcd_sex)
table(allfixed2$rcd_any)
table(allfixed2$rcd_vio)
table(allfixed2$rcd_sexc)


#class 3.
allfixed3 <- filter(ecwc2, include == 0, classID == 3, fixedrecidSex == 1,
                 fixedrecidAny == 1, fixedrecidSexC == 1,
                 fixedrecidVio == 1)
```

```
recid3_allfixed <- data.frame(x1 = allfixed3$rcd_sex,
                              x2 = allfixed3$rcd_any,
                              x3 = allfixed3$rcd_vio,
                              x4 = allfixed3$rcd_sexc)
describe(recid3_allfixed)

FUrecid3_fixed <- data.frame(x1 = allfixed3$TimeSex,
                             x2 = allfixed3$TimeAny,
                             x3 = allfixed3$TimeVio,
                             x4 = allfixed3$TimeSexC)
FUrecid3descrip_fixed <- describe(FUrecid3_fixed)

#descriptives.
mean(FUrecid3descrip_fixed$mean)
mean(FUrecid3descrip_fixed$sd)

#frequencies.
table(allfixed3$rcd_sex)
table(allfixed3$rcd_any)
table(allfixed3$rcd_vio)
table(allfixed3$rcd_sexc)
```
